# Prenatal mobile applications reported in the scientific literature: a scoping review

**DOI:** 10.1590/1980-220X-REEUSP-2024-0236en

**Published:** 2025-02-07

**Authors:** Arlane Silva Carvalho Chaves, Jhonata Gabriel Moura Silva, Layane Mota de Souza de Jesus, Wilza Carla Spiri, Rodrigo Jensen

**Affiliations:** 1 Universidade Estadual Paulista “Júlio de Mesquita Filho”, Faculdade de Medicina de Botucatu, Programa de Pós-graduação em Enfermagem, Botucatu, SP, Brazil.; 2 Universidade Federal do Maranhão, Centro de Ciências de Imperatriz, Departamento de Medicina, Imperatriz, MA, Brazil.; 3 Universidade de São Paulo, Escola de Enfermagem, São Paulo, SP, Brazil.

**Keywords:** Mobile Applications, Prenatal Care, eHealth Strategies, Review, Nursing Informatics, Aplicaciones Móviles, Atención Prenatal, Estrategias de eSalud, Revisión, Informática Aplicada a la Enfermería

## Abstract

**Objective:**

To map the prenatal mobile applications described and/or evaluated in the scientific literature.

**Method:**

Scoping review, based on JBI recommendations, with a time frame from 2017 to 2022. The searches were carried out in October/2023 in the following databases/portals: Lilacs via BVS, Web of Science, MEDLINE, Cochrane Database of Systematic Reviews, Scopus, CINAHL, and the Thesis and Dissertation Bank of the Coordination for the Improvement of Higher Education Personnel (CAPES).

**Results:**

Forty-nine studies were analyzed, including 45 articles, three dissertations and one thesis, published in Portuguese and English. The applications are intended for health education/self-care (46.9%), clinical monitoring (28.5%), for use by health professionals (16.3%), research/data collection (6.1%) and professional education (2%).

**Conclusion:**

The applications described and/or evaluated in the studies are diverse in their purposes and public and confirm the inclusion of mobile technology in the care and health monitoring of pregnant women.

## RESUMEN

**Objetivo:**

Mapear las aplicaciones móviles de prénatal descritas y/o evaluadas en la literatura científica.

**Método:**

Revisión de alcance, basada en recomendaciones del JBI, con un horizonte temporal de 2017 a 2022. Las búsquedas se realizaron en octubre/2023 en las bases/portales Lilacs vía BVS, *Web of Science*, MEDLINE, *Base de datos Cochrane de revisiones sistemáticas*, Scopus, CINAHL y Banco de Tesis y Disertaciones de la Coordinación para el Perfeccionamiento del Personal de Educación Superior (CAPES).

**Resultados:**

Se analizaron 49 estudios, entre ellos 45 artículos, tres disertaciones y una tesis, publicados en portugués e inglés. Las aplicaciones están destinadas a educación sanitaria/autocuidado (46,9%), seguimiento clínico (28,5%), uso de profesionales sanitarios (16,3%), investigación/recopilación de datos (6,1%), y educación profesional (2%).

**Conclusión:**

Las aplicaciones descritas y/o evaluadas en los estudios son diversas en sus propósitos y destinatarios y confirman la inserción de la tecnología móvil en la atención de salud y seguimiento de las mujeres embarazadas.

## INTRODUCTION

Brazil has an estimated population of over 200 million inhabitants, who use around 447 million mobile devices, using them for an average of 3h41min/day. Therefore, the density of mobile devices is of more than two devices per inhabitant and its time of daily use exceeds the world average of 2h22 min/day^(1,2)^. Such data demonstrate how much mobile technology is embedded in the daily lives of Brazilians and this applies equally to health, in which information and communication technologies are increasingly integrated^(1)^.

From this integration, new interdisciplinary and complementary concepts emerge, among which are electronic health (*eHealth*) and its branch, mobile health (*mHealth*), which generally summarize in their definitions the health practices carried out by electronic, wireless and/or mobile devices, such as cell phones and applications^(3,4)^.

In recent years, the healthcare field has been profoundly influenced by the advancement of mobile technologies. The growing penetration of these devices in modern society has exponentially increased the use of mobile applications in the most diverse areas^(5)^.

One of the most explored areas in *mHealth* has been maternal and child health, especially the prenatal period. Studies show the impact of mobile applications on various possibilities in this context, including health education for pregnant women^(6)^, appointment reminders^(7)^, communication with the professional who provides care during pregnancy^(8)^, among others.

Prenatal care is essential in promoting maternal and fetal health, preventing complications, and ensuring healthy development during pregnancy^(9)^. In this sense, mobile technologies present themselves as potentially valuable tools to improve the quality and access to information and prenatal care^(10)^.

Thus, this study seeks to fill a gap in knowledge by mapping the prenatal mobile applications described in the scientific literature and/or evaluated in use. This description is relevant at a time when health technologies are gaining prominence in the global health scenario and review studies on applications have been based on searches in application stores such as the Apple Store and Google Play.

This research benefits pregnant women, healthcare professionals, and app developers by providing recommendations for improving mobile apps. It aims to provide an overview of the applications described and/or evaluated on prenatal care, contributing to informing clinical practices and guiding future technological innovations in the field of prenatal care through mobile applications.

Therefore, the objective of the study was to map the prenatal mobile applications described and/or evaluated in the scientific literature, in a temporal frame.

## METHOD

### Design of Study

Scoping review study conducted based on the recommendations of The Joanna Briggs Institute (JBI)^(11)^ and in accordance with the recommendations of the guideline *Preferred Reporting Items for Systematic reviews and Meta-Analyses extension for Scoping Reviews* (PRISMA-ScR). The study protocol was published in *Open Science Framework*: https://doi.org/10.17605/OSF.IO/8MC96.

### Development of the Research Question

The PCC mnemonic structure was used^(12)^: P (population) – pregnant women and health professionals; C (concept) – description and/or evaluation of mobile applications; C (context) – health activities related to prenatal care. The guiding question was developed: What mobile applications are described and/or evaluated in the scientific literature on health activities related to prenatal care for pregnant women and/or health professionals?

The search was conducted in October 2023.

### Eligibility Criteria

Considering the updates in health policies for pregnant women, a time frame was defined for the study. Studies published from 2017 to 2022, in English, Portuguese or Spanish, available in full and that presented a description and/or evaluation of mobile applications (for smartphone) in the context of prenatal care, for professional health users or pregnant women.

### Research Strategy

The sample was selected from the Coordination for the Improvement of Higher Education Personnel (*CAPES*) journals website, through the VPN (*Virtual Private Network*) service from the Universidade Estadual de São Paulo (Unesp), unespNET network, and in the search for articles in the databases/websites Latin American and Caribbean Literature in Health Sciences (Lilacs) via the Virtual Health Library (VHL), Web of Science (WoS), National Library of Medicine (MEDLINE), Cochrane Database of Systematic Reviews (COCHRANE), Scopus and Current Nursing and Allied Health Literature (CINAHL)(Chart 1).

**Chart 1:** Database search strategy – Botucatu, SP, Brazil, 2023.

Database	Search strategy
**Lilacs (via VHL)**	*((“prenatal care”) AND (“Mobile Applications”) AND (la “en” OR “pt” OR “es”))*
**Web of Science**	*(ALL= (“Mobile Applications”)) AND ALL= (“Prenatal care” )*
**MEDLINE**	*“mobile applications” [All Fields] AND “prenatal care” [All Fields]*
**Cochrane**	*(“Mobile Applications”): ti,ab,kw AND (“Prenatal care”): ti,ab,kw*
**SCOPUS**	*“mobile applications” and “prenatal care”*
**CINAHL**	*(“mobile applications” or apps or “mobile apps”) AND (“prenatal care” or “pre-natal care” or “perinatal care”)*
**CAPES theses and dissertations bank**	*(“prenatal care”) AND (“Mobile Applications”)*

To organize the search strategy, controlled vocabulary terms were selected from the Health Sciences Descriptors (DeCS), Medical Subject Headings Section (MeSH) and CINAHL Headings, combined with boolean operators. A search was carried out in the CAPES Theses and Dissertations Database (Chart 1).

### Evidence Source Selection

Research titles and abstracts were read and analyzed to identify those potentially eligible for the study. The next phase involved reading each of the selected surveys in full to: a) confirm the relevance to the review question and, if so, b) extract the data of interest, the final phase.

### Data Extraction, Analysis, and Presentation of Results

The results were presented using summary tables and in a descriptive format, in accordance with the recommendations of the PRISMA-ScR guideline.

**Figure 1:**
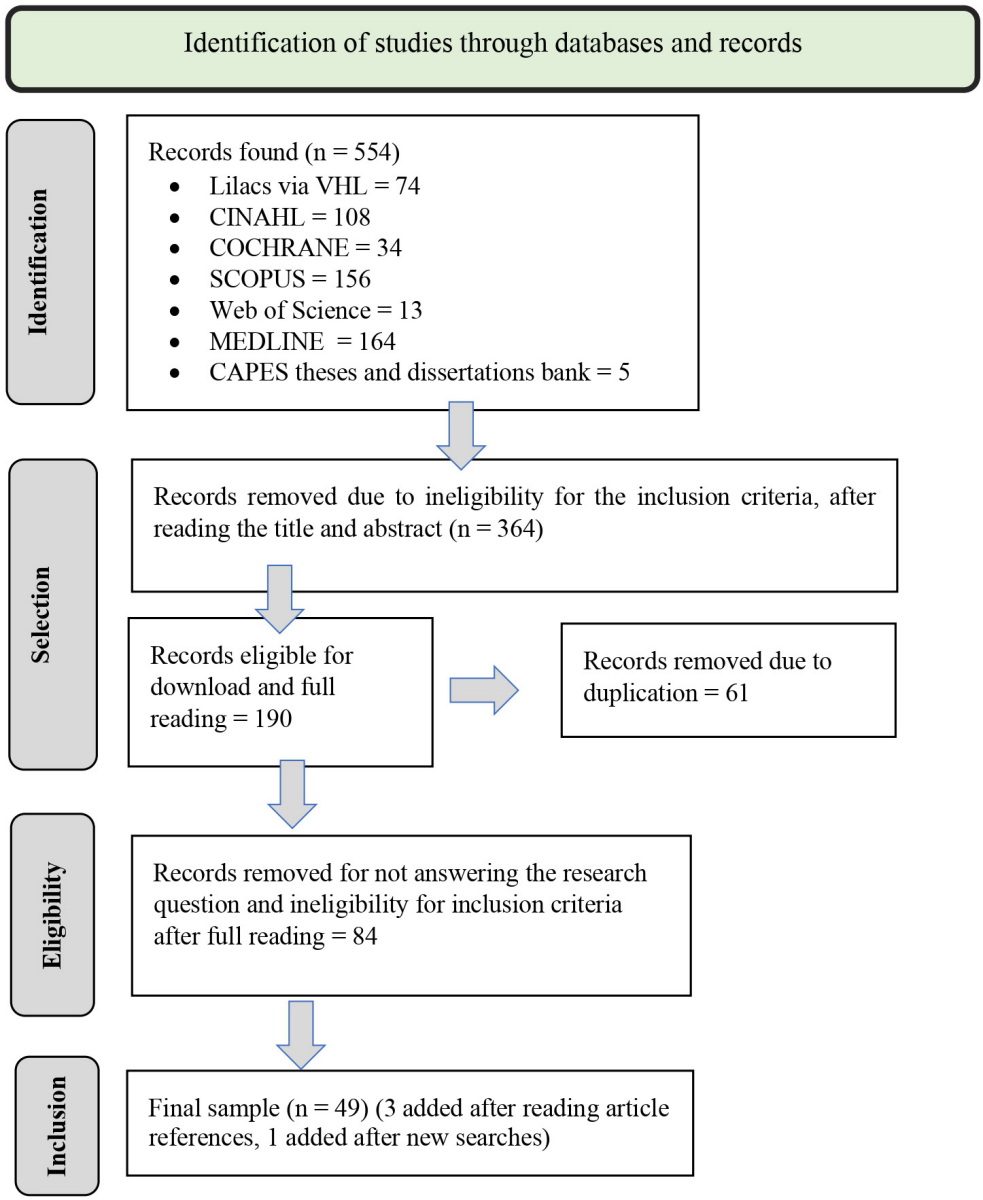
PRISMA-ScR flowchart of study selection and inclusion process in scoping review – Botucatu, SP, Brazil, 2023.

The selection process is presented, according to the PRISMA-ScR flowchart^(13,14)^(Figure 1).

The extraction was conducted by two reviewers, cases of disagreement were resolved by a third reviewer. For data extraction, an instrument adapted from the form recommended by JBI was used^(11)^, with the information: Author/year/country/nature of study, Title/objective of work, Name of application/language/operating system/situation (under development or in use), Central theme of the application/target audience.

The data extracted were mapped using the software Atlas.ti^(15)^, for organizing information and analytical process. These were expressed in summary charts, discussed descriptively, and compared to the findings of other related works.

According to Figure 1, 554 studies were retrieved (549 articles, 2 dissertations, and 3 theses). Titles and abstracts were read, 364 (362 articles and 2 theses) were excluded, and 190 works were downloaded (187 articles: Lilacs via VHL – 34, SCOPUS – 50, MEDLINE – 28, WoS – 6, CINAHL – 44, and COCHRANE – 25; 1 thesis and 2 dissertations), eligible for full reading. After excluding duplicates (61), 129 works were read in full (126 articles, 2 dissertations, and 1 thesis).

At this stage, 84 studies were excluded because they did not deal with applications for use in smartphones or addressed interventions using applications already described in other works analyzed/included in the review. The reference list of the included studies was also analyzed (3 articles and 1 dissertation were added).

## RESULTS

Once the criteria have been applied, after reading and critically analyzing the publications, 49 studies were included, being 45 articles, 3 dissertations and 1 thesis, published between 2017 and 2022, predominantly in English (79.6%) and Portuguese (20.4%). Regarding the country of origin, they were developed by researchers from the Americas (44.8%) (Brazil 11, Canada 1, USA 10, Dominican Republic 1), Asia (24.4%) (Indonesia 1, China 1, Singapore 1, Japan 1, India 1, Iran 2, Israel 1, Malaysia 1, Oman 1, Republic of Korea 1, Taiwan 1), Europe (20.4%) (Germany 1, United Kingdom 2, Netherlands 2, Denmark 1, Ireland 1, United Kingdom 1, Italy 1, Norway 1), Africa (6.1%) (Nigeria 1, Kenya 1, Tanzania 1), and Oceania (2%) (Australia 1).

The following research designs were observed: randomized clinical trials (22.4%), methodological studies (24.4%), descriptive studies (20.4%), clinical study protocols (8.1%), cohort studies (8.1%), non-randomized clinical trials (4%), and other research designs (12.2%) (experimental, exploratory, mixed methods, applied, observational, qualitative and quantitative studies).

Conceptual categories were organized according to the purpose of the mobile application presented in the studies: health education/self-care (46.9%), clinical monitoring (28.5%), for use by professionals (16.3%), research/data collection (6.1%), and professional education (2%).

The results of the studies analyzed are presented in Charts by characteristics of mobile applications for the purposes of: literacy, self-care and health education for pregnant women and intervention (Chart 2); clinical monitoring (follow-up, registration, telemonitoring, consultation, monitoring of pathologies and clinical conditions – gestational diabetes mellitus, syphilis, prematurity, psychological aspects, etc.) and intervention (Chart 3); use by health professionals, related to the care of pregnant women (Chart 4); and research/data collection or recruitment of pregnant women for research, intervention, and professional education (Chart 5).

**Chart 2:** Characteristics of mobile applications for literacy, self-care, and health education purposes for pregnant women and intervention – Botucatu, SP, Brazil, 2023.

Author/year/country Nature of the study	Title/objective	Application name/language/operating system/status (under development or in use)	Main theme of the application/target audience
Kim et al., 2018^(16)^, United States, Methodological research/technological production.	*Smartphone-based prenatal education for parents with preterm birth risk factors*/To develop an educational mobile application for future parents diagnosed with risk factors for premature birth, use the mobile application, and test the viability of the concept in a group of expectant parents diagnosed with risk of premature birth.	*Preemie Prep for Parents* (P3)/English/Android and iOS/Development and availability of use for evaluation	Health education/Parents at risk of premature birth
Silva et al., 2019^(17)^, Brazil Descriptive study.	Cross-cultural adaptation of the application *Zero Mothers Die* for mobile devices in Brazil: contributions to digital health with approaches to care centered on e-pregnant woman/To describe the process of adapting the application to combat maternal mortality *Zero Mothers Die*, developed in Europe, in the Brazilian context, with a methodology of individualized listening to pregnant women and mothers who use a high-complexity and teaching reference hospital.	*Zero Mothers Die/*English and French/Android/Being used (Cross-cultural adaptation for Brazil)	Health education/Pregnant women, mothers of children aged 0 to 1 year and health professionals.
Cawley et al., 2020^(18)^, United States, Retrospective cohort study.	*Effect of a Health System-Sponsored Mobile App on Perinatal Health Behaviors: Retrospective Cohort Study*/To evaluate whether the use of the application *Circle* during the prenatal period was associated with better health outcomes and health behaviors among pregnant and postpartum women.	*Circle*/English/iOS/Being used	Evidence-based pregnancy and childcare/Pregnant women and users of *Providence St. Joseph*.
Souza et al., 2021^(8)^, Brazil Randomized controlled clinical trial.	Effectiveness of a mobile application on pregnant women’s adherence to prenatal consultations: randomized clinical trial/To evaluate the effectiveness of a mobile application for cell phones on pregnant women’s adherence to prenatal consultations.	Healthy Pregnancy/Portuguese/Android/Being used	Educational content in a gestational context/Pregnant women and health professionals.
Kennely et al., 2018^(19)^, Ireland Randomized controlled trial.	*Pregnancy Exercise and Nutrition With Smartphone Application Support*/To evaluate the effect of a healthy lifestyle package (an antenatal behavior change intervention supported by smartphone app technology) on the incidence of gestational diabetes mellitus in overweight and obese women.	*Healthy lifestyle package*/English/Android and iOS/Being used	Exercise and nutrition during pregnancy/Pregnant women
Halili et al., 2018^(20)^, Canada, Descriptive qualitative research.	*Development and pilot evaluation of a pregnancy-specific mobile health tool: a qualitative investigation of SmartMoms Canada/*To conduct a preliminary exploration of women’s attitudes towards the app *SmartMoms Canada* as a reliable source of information and ability to address lifestyle behaviors related to gestational weight gain.	*SmartMoms Canada*/English and French/Android and iOS/Under development	Monitoring gestational weight gain/Pregnant women
Bush et al., 2017^(21)^, United States, Case Study.	*Impact of a Mobile Health Application on User Engagement and Pregnancy Outcomes Among Wyoming Medicaid Members*/To assess whether a mobile application can align patient behavior around prenatal care with best practices, thereby improving pregnancy outcomes.	WYhealth Due Date Plus/English/ -------- Being used	Screening for gestational risk factors and care/Pregnant women
Nørgaard et al., 2017^(22)^, Denmark, Cohort Study.	*Use of the smartphone application Pregnant with Diabetes*/To evaluate the use of the application usage *Pregnant with Diabetes* locally, nationally and internationally.	*Pregnant with Diabetes*/English/ Android and iOS/--------	Health education for pregnant women with diabetes/ Pregnant women with pre-existing diabetes
van Beukering et al., 2019^(23)^, Netherlands, Mixed methods study.	*Usability and Usefulness of a Mobile Health App for Pregnancy-Related Work Advice: Mixed-Methods Approach*/Evaluate the usability of the application *Pregnancy and Work* and perceived usefulness.	*Pregnancy and Work/* Dutch and English/------- Under development (Usability evaluation)	Work advice during pregnancy/Pregnant women
Shorey et al., 2019^(24)^, Singapore, Mixed methods study.	*Effectiveness of a Technology-Based Supportive Educational Parenting Program on Parental Outcomes (Part 1): Randomized Controlled Trial*/To evaluate the effectiveness of a technology-based supportive educational parenting program (SEPP) on parenting outcomes during the perinatal period in couples.	SEEP/ English/ ---------/--------	Parental educational program intervention, Health education and monitoring/Couples
Kawasaki et al., 2021^(25)^, Japan Randomized controlled study protocol.	*Protocol for an interventional study to reduce postpartum weight retention in obese mothers using the internet of things and a mobile application: a randomized controlled trial (SpringMom)*/To assess whether weight change in obese women at 12 months postpartum compared to before pregnancy becomes lower with an intervention using devices with internet of things and mobile applications during pregnancy up to 1 year postpartum compared to and without intervention.	*Ninsanpu Plus Breast Callus*/ ----- Android and iOS/ Being used	Monitoring gestational weight gain/Pregnant and postpartum women
Carrilho et al., 2019^(26)^, Brazil, Observational, exploratory, and descriptive study.	Pregnant Women’s Perception of the Birth Plan Interface in the Application “Meu Pré-Natal”: Observational Validation Study/To assess the perception of pregnant women regarding the communicability of preparing a birth plan through a mobile application.	*My prenatal care*/ English, Portuguese and Spanish/Android iOS/Being used	Health education in prenatal, childbirth and postpartum period/Pregnant women
Moulaei et al., 2021^(27)^, Iran, Descriptive-applied study/technological production.	*The Development and Usability Assessment of an mHealth Application to Encourage Self-Care in Pregnant Women against COVID-19*/To develop and evaluate a self-care app to provide self-care for pregnant women against COVID-19.	*Self-Care in Pregnant Women against COVID-19*/English/----------- Under development (prototyping)	Self-care during pregnancy against COVID-19/Mothers and pregnant women
Yee et al., 2021^(28)^, United States, Prospective and qualitative study.	*Patient and Provider Perspectives on a Novel Mobile Health Intervention for Low-Income Pregnant Women With Gestational or Type 2 Diabetes Mellitus*/To collect feedback from SweetMama patients and healthcare professionals, who would inform the necessary modifications to improve the usability, functionality and accessibility of the application.	*SweetMama*/English/----------- development (prototype usability evaluation)	Health education on type 2 diabetes mellitus and gestational diabetes/Pregnant women with Gestational Diabetes Mellitus and Type 2 Diabetes Mellitus
Lee et al., 2022^(29)^, Republic of Korea, Prospective and qualitative study.	*Self-Care Mobile Application for South Korean Pregnant Women at Work: Development and Usability Study*/To develop a mobile-based intervention application for Korean pregnant women at work and test its usability and preliminary effects to enhance their self-care practices.	SPWW/English/ Android/Development and usability	Self-care during pregnancy/Pregnant women
Al Hashmi et al., 2022^(30)^, Oman, Clinical Trial.	*Development, feasibility and acceptability of a self-efficacy-enhancing smartphone application among pregnant women with gestational diabetes mellitus: single-arm pilot clinical trial*/To assess the feasibility and acceptability of an application for *smartphone*, SEESPA, among pregnant women with gestational diabetes mellitus.	SEESPA/ Arabic and English/ Android and iOS/ Under development	Management and control of gestational diabetes mellitus/Women with gestational diabetes mellitus
Sidik et al., 2020^(31)^, Malaysia, Cluster-randomized controlled clinical trial protocol.	*KEPT-app trial: a pragmatic, single-blind, parallel, cluster-randomized effectiveness study of pelvic floor muscle training among incontinent pregnant women: study protocol*/To evaluate the effectiveness of the KEPT application guidance application in the treatment of urinary incontinence in pregnant women.	KEPT/ ----/ ----/ Under development	Pelvic floor muscle training/Pregnant women and women
Chang et al., 2022^(32)^. Taiwan Randomized controlled study.	*Efficacy of a Smartphone Application to Promote Maternal Influenza Vaccination: A Randomized Controlled Trial*/To evaluate the effectiveness of the application to promote maternal vaccination against influenza.	*Smartphone Application to Promote Maternal Influenza Vaccination*/ ----/ ----/ Being used	Flu vaccination/Pregnant women
Dudley et al., 2019^(33)^, United States, Randomized controlled trial.	*Factors associated with referring close contacts to an app with individually-tailored vaccine information*/To determine which factors are associated with a greater likelihood of pregnant women referring their close contacts to this educational application.	*MomsTalkShots* ----/ ----/ Use for intervention	Maternal and child vaccination/Pregnant women
Karamolahi et al., 2021^(34)^, Iran Randomized clinical trial.	*Efficacy of mobile app-based training on health literacy among pregnant women: A randomized controlled trial study*/To determine the effect of mobile application-based training on health literacy among pregnant women.	-------/ ------- Android/ Being used	Health education/Pregnant women
Queiroz et al., 2021^(35)^, Brazil, Applied, methodological nature with a qualitative approach.	Evaluation of the application “GestAção” from the perspective of semiotics: the perspective of pregnant women/Evaluate the application GestAção, from the perspective of pregnant women, in the light of Semiotics.	GestAção/ Portuguese Android/ Use for evaluation	Pregnancy monitoring/Pregnant women
Barbosa, 2021^(36)^, Brazil, Technological development.	Development of a Mobile Application for Monitoring Pregnant Women During Prenatal Care, with an Emphasis on Covid-19/To develop a mobile application for monitoring pregnant women during prenatal care, with an emphasis on COVID-19.	INFOGEST in Prenatal Care/ Portuguese/Android/Under development	Health education for pregnant women and COVID-19 during pregnancy/Pregnant women
Chiodi, 2020^(37)^, Brazil, Applied research, methodological design.	*m-health* technology development and evaluation aimed at pregnant women at risk of preterm birth: an expression of designer thinking*.*/To develop and evaluate an application prototype aimed at pregnant women at risk of preterm birth.	Will it be premature?/ Portuguese/ Android and iOS/ Under development	Risk for preterm birth/Pregnant women

**Chart 3:** Characteristics of mobile applications for clinical monitoring and intervention purposes – Botucatu, SP, Brazil, 2023.

Author/year/country Nature of the study	Title/objective	Application name/language/operating system/status (under development or in use)	Main theme of the application/ target audience
Miremberg et al., 2018^(38)^, Israel, Randomized controlled trial	*The impact of a daily smartphone-based feedback system among women with gestational diabetes on compliance, glycemic control, satisfaction, and pregnancy outcome: a randomized controlled trial*/To analyze the impact of introducing a smartphone-based daily feedback and communication platform between patients with gestational diabetes mellitus and their physicians, on patient adherence, glycemic control, pregnancy outcome and patient satisfaction.	*Glucosebuddy*/ English/ Android and iOS/ Being used	Blood glucose monitoring/People with diabetes
Silva et al., 2021^(39)^, Brazil, Technological Production.	Web application for monitoring pregnant and postpartum women: technological production/To develop the production process of a web application prototype for monitoring pregnant and postpartum women.	Gestar *care*/ Portuguese/ Android and iOS/ Under development and in use	Maternal and child health and online consultation/Health professionals, pregnant women and postpartum women
Doherty et al., 2018^(40)^, Ireland and the United Kingdom, Clinical Research.	*A Mobile App for the Self-Report of Psychological Well-Being During Pregnancy (BrightSelf): Qualitative Design Study*/To explore the issues and challenges involving the use of mobile phones for self-reported psychological well-being during pregnancy.	*BrightSelf*/ English/ Android and iOS/ Being used	Assessment and monitoring of depression and mood during prenatal care/Pregnant women
Hantsoo et al., 2018^(41)^, United States, Randomized controlled trial.	*A Mobile Application for Monitoring and Management of Depressed Mood in a Vulnerable Pregnant Population*/To evaluate the impact of a mobile mood tracking and alerting application on patient engagement and health care delivery in an obstetric setting among low-income racial and ethnic minority women experiencing depressive symptoms.	*Mood Tracking and Alert* (MTA)/ English/ Android and iOS/ Being used	Mental health/General population
Marko et al., 2019^(42)^, United States, Controlled Study.	*A Mobile Prenatal Care App to Reduce In-Person Visits: Prospective Controlled Trial*/To test the effectiveness of a prenatal care mobile app to facilitate reduced in-person visit scheduling for low-risk pregnancies while maintaining patient and provider satisfaction.	*Babyscripts*/ English/ iOS/ Being used	Educational content/ Pregnant women
Sales et al., 2019^(43)^, Brazil, Methodological research.	Development and evaluation of an application for the control of syphilis in pregnant women/To develop and evaluate an application for the control of syphilis in pregnant women.	SELP/ Portuguese/ Android and iOS/Under development	Syphilis prevention/General population and health professionals
Borgen et al., 2019^(44)^, Norway, Randomized controlled clinical trial.	*Effect of the Pregnant+ smartphone application in women with gestational diabetes mellitus: a randomized controlled trial in Norway*/To evaluate the effect of the application *Pregnant+* on the 2-hour glucose level of the routine postpartum oral glucose tolerance test in women with gestational diabetes mellitus.	*Pregnant+*/ ------- English/ Being used	Management of gestational diabetes/Pregnant women
Xydopoulos et al., 2019^(45)^, United Kingdom, Economic viability - Cohort study.	*Home blood-pressure monitoring in a hypertensive pregnant population: cost-minimization study*/To conduct an economic analysis of health of the application HBPM compared to traditional monitoring in hypertensive pregnant women.	HBPMI*/* English/ -----/ Under development	Health education and health monitoring/Pregnant women
Muller et al., 2020^(46)^, Germany, Randomized controlled clinical trial protocol.	*Effectiveness and cost-effectiveness of an electronic mindfulness-based intervention (eMBI) on maternal mental health during pregnancy: the mindmom study protocol for a randomized controlled clinical trial*/To evaluate the clinical effectiveness and cost-benefit of an application developed by an interdisciplinary team of gynecologists, psychologists and midwives, teaching pregnant women how to deal with stress, pregnancy-related anxiety, and depressive symptoms.	*Mindmom*/ ----------- ----------- Being used	Maternal mental health/Pregnant women
Cramer et al., 2018^(47)^, United States, Experimental study - Community-based participatory research.	*The feasibility and promise of mobile technology with community health worker reinforcement to reduce rural preterm birth*/To assess the feasibility of a smartphone platform intervention combined with community health worker reinforcement among rural pregnant women; to obtain data on the intervention on birth outcomes, patient activation, and adherence to medical care; and to assess the financial implications of the intervention using return on investment	PTP/ English and Spanish/ -------/ Under development (feasibility study – pilot)	Pregnancy health education/Pregnant women
van den Heuvel et al., 2019^(48)^, Netherlands, Observational Prospective.	*SAFE@HOME - Feasibility study of a telemonitoring platform combining blood pressure and preeclampsia symptoms in pregnancy care*/To assess patient acceptability of telemonitoring using the application and blood pressure monitor and to review internal infrastructure to analyze all observed measurements.	Luscii/ Dutch/ iOS/ Being used	Telemonitoring of blood pressure during prenatal care/Pregnant women and health professionals
Zuccolo et al., 2021^(49)^, Brazil, Randomized clinical trial protocol.	*A smartphone-assisted brief online cognitive-behavioral intervention for pregnant women with depression: a study protocol of a randomized controlled trial*/To evaluate the effectiveness of *Motherly*, for cognitive-behavioral therapies in the treatment of maternal depression.	*Motherly*/ Portuguese Android/ Being used	Health education for pregnant women and babies (mental health – behavioral activation)/Pregnant women and mothers
Musyoka et al., 2019^(50)^, Kenya, Methodological, exploratory experimental study.	*A 24-hour ambulatory blood pressure monitoring system for preeclampsia management in antenatal care*/To implement a 24-hour outpatient blood pressure monitoring system for pregnant women using a smartwatch in conjunction with a mobile application, to assist in the management of pre-eclampsia, based on an Internet of Things architecture.	*Blood pressure monitor*/ English/ Android/ Under development	Arterial pressure monitoring/Pregnant women and professionals

**Chart 4:** Characteristics of mobile applications for use by health professionals involved in the care of pregnant women and intervention –Botucatu, SP, Brazil, 2023.

Author/year/country Nature of the study	Title/objective	Application name/language/operating system/status (under development or in use)	Main theme of the application/target audience
Gbadamosi et al., 2018^(51)^, Nigeria, Demonstration study.	*A Patient-Held Smartcard With a Unique Identifier and an mHealth Platform to Improve the Availability of Prenatal Test Results in Rural Nigeria: Demonstration Study*/To describe the development and implementation of an *mHealth* platform integrated and capable of capturing health information, including laboratory test results, and encrypting it on a patient’s smart card, which can be read at the point of delivery without the need for an Internet connection.	*Vitira Health*/ English/Android/Development (restricted to authorized users)	Smartcard QR code reading and display of prenatal exam results/Authorized patients
Borsari et al., 2018^(52)^, Italy, Feasibility study.	*An Innovative Mobile Health System to Improve and Standardize Antenatal Care Among Underserved Communities: A Feasibility Study in an Italian Hosting Center for Asylum Seekers*/To present the results of a pilot study evaluating the functionality, feasibility, and acceptance rate of the mobile health system in providing antenatal care to a migrant population hosted within the largest European reception center.	PANDA/ English and French/ Android/ Under development	Prenatal care focused on the Diagnostic Assessment of Pregnancy and the Newborn/Health professionals (doctors, nurses, midwives, community health agents)
Shah et al., 2019^(53)^, India. Feasibility study.	*High uptake of an innovative mobile phone application among community health workers in rural India: An implementation study*/To assess the acceptance, feasibility and effectiveness of ImTeCHO in the personal work environment.	ImTeCHO/ Indian/Android/ Being used	Work management in monitoring pregnant women and children/Professionals, *Accredited social health activist* - ASHA (similar to community health workers)
Bonnell et al., 2018^(54)^, Dominican Republic, Feasibility study.	*Community Health Workers and Use of mHealth: Improving Identification of Pregnancy Complications and Access to Care in the Dominican Republic*/To assess the feasibility and acceptability of using mobile health technology by community health workers to improve the identification of pregnancy complications and access to care for pregnant women.	*Poimapper* (mobile application and data management portal)/------/Android and iOS/In use	Identification of pregnancy complications and access to care for pregnant women/Professionals, Community health workers
Hackett et al., 2018^(55)^, Tanzania, Cluster randomized trial.	*Impact of smartphone-assisted prenatal home visits on women’s use of facility delivery: Results from a cluster-randomized trial in rural Tanzania*/To assess the impact of an application for Community Health Workers on women’s use of childbirth services in rural Tanzania.	SUSTAIN/ ----/ ----/ Being used	Management and monitoring of home visits by pregnant women/Community Health Agents (*ACS*s)
Liu and Wang, 2021^(56)^, China, Clinical intervention study.	*Application of Smart Mobile Medical Services in Maternal Health Care Management*/To standardize the management of pregnant women’s health, improve the health level of pregnant women and improve the outcome of pregnancy with the help of the mobile application.	*Health Assistant*/ ------- ------- Being used	Health education management/Pregnant woman
Delmaifanis et al., 2021^(57)^, Indonesia, Conceptual study and application development.	*mHealth Conceptual Model for Providing Quality Antenatal Care in Health Centers during the Coronavirus Disease 2019 Pandemic*/To build the concept of *mHealth* to improve the quality of prenatal care, to add resources that support service workers during the COVID-19 pandemic.	-------/ English/ ------- Under development	Prenatal care and monitoring during the pandemic/Midwives, physicians, and pregnant women
Fonseca, 2020^(58)^, Brazil, Documentary and methodological study.	To review and update of ICNP® Terminology Subsets for Women’s Health, Prenatal and Postpartum Care and Proposal for Building an Application for Mobile Devices/To describe the review and update process and present the terminology subsets for Women’s Health and Prenatal and Postpartum Care, according to ICNP® 2017 and propose the modeling for building an application for mobile devices, to make the Terminology Subsets available.	ICNP-PHC/ Portuguese/ Android and iOS/ Under development	Terminological subsets of Women’s Health and Prenatal and Postpartum, according to ICNP®/Nurses, nursing professors, and nursing students

**Chart 5:** Characteristics of mobile applications for research/data collection or recruitment of pregnant women for research, intervention and teaching in obstetric nursing – Botucatu, SP, Brazil, 2023.

Author/year/country Nature of the study	Title/objective	Application name/language/operating system/status (under development or in use)	Main theme of the application/target audience
Krishnamurti et al., 2021^(59)^, United States, Prospective cohort study.	*Use of a Smartphone App to Explore Potential Underuse of Prophylactic Aspirin for Preeclampsia*/To assess the potential for underutilization of low-dose aspirin treatment and the reasons for underutilization using data from a prenatal app.	*My Healthy Pregnancy* (MHP)/ English/ Android and iOS/ Being used	Educational content in a gestational context/Pregnant women
Vignato et al., 2019^(60)^, United States, Cross-sectional study.	*Using Mobile Health Applications for the Rapid Recruitment of Perinatal Women/*To describe how two US research teams used a mobile app aimed at pregnant women to recruit participants for a cross-sectional study and a clinical trial.	*Ovia Pregnancy*/ English/Android and iOS/ Being used	Pregnancy health education/Pregnant women
Keedle et al., 2018^(61)^, Austrália, Qualitative research	*The Design, Development, and Evaluation of a Qualitative Data Collection Application for Pregnant Women*/To describe the development and evaluation of a mobile application to collect qualitative data.	*myVBACapp* English/ Android and iOS/ Under development	Data collection/Women
Amorim, 2022^(62)^, Brazil, Methodological Study.	GRAVIDAPP 2.0: educational application for the practice of obstetric nursing in higher education/To develop an educational application entitled *gravidapp 2.0* for theoretical and practical support for students of the Obstetric Nursing (EO) course at the Magalhães Barata School of Nursing at the Universidade Estadual do Pará (EEMB-UEPA).	Gravidapp 2.0/ Portuguese/ Android/ Being used	Obstetric Nursing Teaching/ Students, residents and/or professionals in Obstetric Nursing

As to applications for purposes of literacy, self-care and health education for pregnant women (Chart 2), it was identified that four of these were developed to promote prenatal care or even for specific conditions such as COVID-19 and syphilis. Eight (16.3%) applications were used for intervention, either in the evaluation of use in relation to monitoring of clinical conditions (weight gain, COVID-19), adherence to consultations, and encouragement of self-care with information on a healthy lifestyle.

Six (12.2%) applications were evaluated, either globally or in a specific function, regarding the acceptability of their use for telemonitoring and regarding the collection of feedback to improve the application functionality and accessibility. Three (6.1%) were evaluated for effectiveness in promoting treatments, adherence to vaccines, best practices in prenatal care and scheduling of appointments. One (2%) application for fighting mortality was in the process of cross-cultural adaptation for Brazil and one (2%) was evaluated for content quality.

Chart 3 presents studies on clinical monitoring applications. Three (6.1%) studies were identified regarding the development of applications aimed at monitoring and controlling syphilis, and one identified requirements for the ideal application. Six (12.2%) studies carried out intervention using applications and evaluated several aspects: patient-professional communication, potential for monitoring (gestational diabetes mellitus, postpartum oral glucose tolerance, psychological aspects, blood pressure combined with smartwatch) and the effect of using mobile technology in monitoring combined with reinforcement of Community Health Agents (*ACS*s).

Three (6.1%) studies evaluated the effectiveness of scheduling and delivering brief cognitive-behavioral therapies to treat maternal depression. Two other studies (4%) evaluated the cost-benefit of applications when compared to traditional monitoring and clinical effectiveness, and finally one aimed to evaluate the acceptability of an application for telemonitoring blood pressure.

In Chart 4, regarding applications for use by professionals, two (4%) studies indicated the development of an application to capture health information, access exams, and to assist care during the COVID-19 pandemic. Three (6.1%) evaluated the functionality and viability of the applications when they were used to provide prenatal care or to assist the work of community health workers. One (2%) study evaluated the impact of implementing the application on birth outcomes, through its use for communication between *ACS*s and the health team, which directed care. Two other studies (4%) standardized assistance, both through guidance provided by the application and through standardization of the language used among professionals.

Chart 5 presents three (6.1%) studies addressing applications developed or in use as an accessible means to support data collection for scientific research involving pregnant women, and one (2%) study demonstrated the development and use of an application for professional education.

## DISCUSSION

Mobile applications have supported several health activities, in the context of prenatal care, assisting in health literacy, self-care, clinical monitoring by the user and health professionals, as well as for nursing education purposes.

Health literacy is potentially facilitated by the support of applications, whose use has been shown to be positive for parental education in prenatal care for pregnant women at risk of premature birth^(16)^, identification of the need to address psychosocial aspects of high-risk pregnancy and preparation for possible hospitalization, preterm birth and neonatal hospitalization^(37)^, promotion of self-care during the COVID-19 pandemic, as well as provision of information, and guidance on medications and complications of COVID-19 during pregnancy^(27,36)^.

Intervention studies have shown that the use of applications was effective for monitoring clinical conditions, promoting communication between the healthcare team and pregnant women^(8)^, in the adherence of pregnant women to prenatal consultations, improvement of healthy practices, self-care during prenatal care^(8,18,25)^), health literacy of pregnant women in the context of the pandemic^(34)^, and personalized vaccine information^(33)^.

Regarding the applications already developed that were being evaluated globally or in any of its functions, the studies showed positive usability and acceptability. They showed positive use for telemonitoring and provision of information on glycemic index, via technology, in rural areas and in developing countries^(22)^, facilitating maternal and child care in an interactive and educational way^(35)^, with high clinical efficacy in maternal mental health, promoting psychological support for fathers and mothers, through reliable sources of information^(24,30)^ and good evaluation perceived even when only the interface for the birth plan was evaluated^(26)^.

Studies^(23,26)^ analyzed feedback from users and identified that the design used influences their interaction with the application. Positive results were found in the use of interactive interfaces that offer content with information to the user.

Studies have evaluated the effectiveness of applications in terms of reminding pregnant women about influenza vaccination and improving their knowledge about the vaccine, promoting positive attitudes towards vaccination^(32)^. Furthermore, Sidik^(31)^ proposed the development of an application according to an intervention mapping, evaluated for effectiveness in providing guidance for the treatment of urinary incontinence in pregnant women.

The application *SweetMama* was implemented as a mobile health intervention for low-income pregnant women with gestational diabetes, and has been shown to be easy to use, efficient, and potentially impactful, designed to meet the unique needs of this population^(28)^.

In addition to the development and evaluation of applications, a study^(34)^ proposed the cultural adaptation of an application to combat maternal mortality, from English to Brazilian Portuguese. *Zero Mothers Die* is an application with information about the prenatal routine of health facilities with teaching activities, which can provide channels for dialogue with pregnant women and updating of professionals in training^(17)^.

The concern with the clinical monitoring of certain conditions that may emerge during pregnancy or that affect the pregnant woman directly or indirectly was highlighted in studies, both in development proposals and/or clinical trial protocols, and in interventions using such tools^(46,49)^.

A study should be highlighted^(43)^ in which the authors developed the application SELP with information in text and video about the signs, symptoms, causes, risks, and treatment for syphilis. The technology applies mapping resources to locate health centers, creates alerts for pregnant women and their partners about treatment dates, and maps the contact network of syphilis carriers anonymously^(43)^.

Mobile applications are an important tool for optimizing time and shortening distances. Because they are easy-to-access and quick-to-consult tools, they have proven to be effective in reducing in-person visits, favoring virtual care for pregnant and postpartum women, and reducing waiting times for emergency services and professionals, given their communication resources^(39,42)^. In addition to the possibility of communicating with a professional for care, several studies demonstrate the effectiveness of applications in telemonitoring blood pressure (BP) in pregnant women^(45,48,51)^.

It was possible to promote BP monitoring in pregnant women using a smartwatch in conjunction with a mobile application; there was a reduction in the number of antenatal outpatient visits for BP-related reasons compared to management according to local guidelines and cost savings with the use of the application^(45,51)^. The solution has demonstrated potential for adoption in healthcare systems in developing countries, given the application simplicity and accessibility^(51)^.

Another feature, of sending alerts to start repeated BP measurements, combined with alerts for self-reported symptoms of pre-eclampsia, also demonstrated clinical relevance^(48)^.

Daily feedback and communication intervention between patients and healthcare team improved patient adherence and glycemic control, and reduced the rate of insulin treatment^(38)^.

In contrast, when analyzing the effect of using an application for pregnant women (Pregnant+) in women with diabetes mellitus in Norway, the results showed that the use of the application had no effect on the main outcome of glucose monitoring and reduction of rates^(44)^. This finding may be related to the fact that the technology was not developed specifically for this purpose, which may compromise the effectiveness of the telemonitoring strategy.

In terms of impact on perinatal mental health, a tool for self-reporting psychological well-being has shown potential in allowing greater understanding of well-being in pregnancy^(40)^. Another study^(41)^ presented the need to promote cognitive-behavioral therapy using mobile technology, and demonstrated the effectiveness of the application in improving mental health services in obstetric practice. Interventions like this can help in early identification, monitoring, and treatment of women at high risk of developing psychiatric symptoms during pregnancy, besides having the potential to provide information based on scientific evidence, helping them to identify anxieties and insecurities during this period and seek support.

Mobile applications have a development and maintenance cost on digital platforms. Therefore, cost-effectiveness assessment studies are also described.

A study^(47)^ carried out a financial analysis (return on investment) in which a group of pregnant women (intervention group) received a smartphone with internet and access to a personalized prenatal platform (evaluated in the study) with automated text messages, chat function and hyperlinks, and weekly contact from the *ACS*. The control group received usual prenatal care and printed educational materials. The intervention received positive evaluations from users and gained ground in adherence to prenatal care and improvement of birth outcomes in rural communities. The financial impact analysis showed positive cost-effectiveness results for the intervention, producing reduced healthcare costs for the intervention group, compared to the control group, with an overall average savings difference of US$1,079 for intervention participants.

Given the versatility and accessibility of the applications, they were also used as resources, for easy and quick access to recruit participants for scientific research involving pregnant women^(59)^. Using applications for participant recruitment allows for real-time data and instant access to data, as well as having the ability to contact people from multiple locations^(61)^, a promising strategy for recruiting large and diverse samples^(60)^.

The versatility of applications has allowed their use to be integrated with other health tools or platforms, to implement prenatal care and access information about pregnant women, even in places with low internet availability^(61)^ or during the COVID-19 pandemic^(57)^, optimizing the quality and effectiveness of services and promoting health.

In addition to helping to assist pregnant women, applications have been used by health professionals to provide prenatal care recommendations, based on structured guidelines and recommendations. Some results are observed, such as improved communication between patients and caregivers^(52)^, systematization of home visits and qualification of prenatal and postnatal care, together with the care of more complex cases, facilitated by the possibility of contact between the *ACS* and the health team^(53,54)^.

The use of smartphones as a component of intervention packages aimed at safe motherhood can improve the support and effectiveness of *ACS*s^(58)^ and other health professionals’ work, improving patient adherence rates to prenatal exams and follow-up, by providing information during pregnancy and the perinatal period^(56)^.

Besides the applications developed to offer care via mobile phones, a study^(56)^ proposed the construction of an application for mobile devices, to make the Terminological Subset of Women’s Health, Prenatal and Postpartum care available from the Portuguese translated version of the International Classification for Nursing Practice (ICNP®). Another study^(62)^ proposed the development of an application for teaching obstetric nursing.

These proposals demonstrate a new profile of mobile applications aimed at supporting the assistance provided by professionals and helping students in the teaching-learning process of specific skills for obstetric nursing.

### Study Limitations

Limitations of the study should be considered. A time frame from 2017 to 2022 was considered, limiting the analysis of scientific production to a specific period. The methodological quality of the studies was not assessed. Some included studies did not present complete information on the applications and, sometimes, characterization information, such as operating system, language, and status (under development or in use), was missing. It was not possible to assess the scientific basis of the content of the applications with criteria; however, it is worth noting that dissemination in scientific publications makes them more reliable for recommendation/use in prenatal care.

### Contributions to Health-Related Research

This review provides a comprehensive overview of the mobile applications described/evaluated in the scientific literature, in the context of monitoring pregnant women in different clinical situations during the prenatal period. They promote access to information in the context of prenatal care using mobile technology, both for pregnant women and their partners, health professionals and students. Considering the publication of application review studies that start from the analysis of application stores, this review starts from applications published in scientific productions, which allows greater confidence in the recommendations indicated. Furthermore, this study provides recommendations for the development and evaluation of mobile health technologies.

## CONCLUSION

Health technologies are increasingly gaining prominence on the global stage. Therefore, characterizing the mobile applications used or designed to monitor pregnant women based on scientific literature provided a broader view of these technologies and their forms of use.

The studies analyzed brought versatile and varied mobile applications, whose use confirms the potential for the insertion of mobile technology in different care scenarios and in monitoring pregnant women’s health. Furthermore, whether used by professionals, health students or pregnant women themselves, the applications above-mentioned have been widely used in the areas of literacy, self-care, health education for pregnant women, clinical monitoring and intervention, teaching in obstetric nursing, and for data collection or recruitment of pregnant women for research.

Future developments may be necessary so that applications can be evaluated using instruments that consider technical aspects, safety, quality of these solutions or even explore the potential of applications in other interfaces, monitoring pregnant women throughout prenatal care, expanding the results presented in this study.
