# Supplementary material for: Prenatal mobile applications reported in the scientific literature: a scoping review
Source: Rev Esc Enferm USP. 2025 Feb 7;58:e20240236. doi: 10.1590/1980-220X-REEUSP-2024-0236en (PMC11804663; doi:10.1590/1980-220X-REEUSP-2024-0236en)
Supplement: Translation

# Translation: Aplicativos móveis de pré-natal relatados na literatura científica: uma revisão de escopo

**DOI:** 10.1590/1980-220X-REEUSP-2024-0236pt

**Authors:** Arlane Silva Carvalho Chaves, Jhonata Gabriel Moura Silva, Layane Mota de Souza de Jesus, Wilza Carla Spiri, Rodrigo Jensen

**Affiliations:** 1 Universidade Estadual Paulista “Júlio de Mesquita Filho”, Faculdade de Medicina de Botucatu, Programa de Pós-graduação em Enfermagem, Botucatu, SP, Brasil.; 2 Universidade Federal do Maranhão, Centro de Ciências de Imperatriz, Departamento de Medicina, Imperatriz, MA, Brasil.; 3 Universidade de São Paulo, Escola de Enfermagem, São Paulo, SP, Brasil.

**Keywords:** Aplicativos Móveis, Cuidado Pré-Natal, Estratégias de eSaúde, Revisão, Informática em Enfermagem

## Abstract

**Objetivo:**

Mapear os aplicativos móveis de pré-natal descritos e/ou avaliados na literatura científica.

**Métodos:**

Revisão de escopo, a partir das recomendações da JBI, com recorte temporal de 2017 a 2022. As buscas foram realizadas em outubro/2023 nas bases/portais Lilacs via BVS, *Web of Science*, MEDLINE, *Cochrane Database of Systematic Reviews*, Scopus, CINAHL e Banco de Teses e Dissertações da Coordenação de Aperfeiçoamento de Pessoal de Nível Superior (CAPES).

**Resultados:**

Foram analisados 49 estudos, sendo 45 artigos, três dissertações e uma tese, publicados em português e inglês. Os aplicativos possuem finalidade para educação em saúde/autocuidado (46,9%), monitoramento clínico (28,5%), para uso por profissionais de saúde (16,3%), pesquisa/coleta de dados (6,1%) e ensino profissional (2%).

**Conclusão:**

Os aplicativos descritos e/ou avaliados nos estudos são diversos em suas finalidades e público e confirmam a inserção da tecnologia móvel no cuidado e monitoramento em saúde das gestantes.

## INTRODUÇÃO

O Brasil possui população estimada de mais de 200 milhões de habitantes, que colocam em uso cerca de 447 milhões de dispositivos móveis, utilizando-os em uma média de 3h41min/dia. Sendo assim, a densidade de dispositivos móveis é superior a dois aparelhos por habitante e seu tempo de utilização diária supera a média mundial de 2h22 min/dia^(1,2)^. Tais dados demonstram o quão inserida no dia-a-dia dos brasileiros está a tecnologia móvel e isso se aplica de igual maneira à saúde, na qual cada vez mais as tecnologias de informação e comunicação são integradas^(1)^.

A partir dessa integração surgem novos conceitos, interdisciplinares e complementares, dentre os quais estão a saúde eletrônica (*eHealth*) e seu ramo, saúde móvel (*mHealth*), que de maneira geral sintetizam em suas definições as práticas de saúde realizadas por dispositivos eletrônicos, sem fio e/ou móveis, como telefones celulares e aplicativos^(3,4)^.

Nos últimos anos, o campo da saúde tem sido profundamente influenciado pelo avanço das tecnologias móveis. A crescente penetração destes dispositivos na sociedade moderna tem elevado exponencialmente o uso de aplicativos móveis nas mais diversas áreas^(5)^.

Uma das áreas mais exploradas na *mHealth* tem sido a saúde materno-infantil, em especial o período pré-natal. Estudos mostram o impacto de aplicativos móveis nas diversas possibilidades nesse contexto, dentre as quais estão a educação em saúde da gestante^(6)^, lembretes de consulta^(7)^, comunicação com o profissional que realiza o atendimento durante o período gravídico^(8)^, entre outras.

A assistência pré-natal é fundamental na promoção da saúde materna e fetal, prevenção de complicações e garantia de um desenvolvimento saudável durante a gestação^(9)^. Nesse sentido, tecnologias móveis apresentam-se como ferramentas potencialmente valiosas para melhorar a qualidade e o acesso à informação e aos cuidados pré-natais^(10)^.

Assim, busca-se nesse estudo preencher lacuna no conhecimento, mapeando-se os aplicativos móveis de pré-natal descritos na literatura científica e/ou avaliados em uso. Tal descrição faz-se relevante em um momento em que as tecnologias em saúde ganham destaque no cenário da saúde global e estudos de revisão sobre aplicativos tem se baseado na busca a lojas de aplicativos como Apple Store e Google Play.

Essa pesquisa beneficia mulheres grávidas, profissionais de saúde e desenvolvedores de aplicativos, oferecendo recomendações para o aprimoramento dos aplicativos móveis. Propõe-se a fornecer um panorama acerca dos aplicativos descritos e/ou avaliados, sobre pré-natal, contribuindo para informar práticas clínicas e orientar futuras inovações tecnológicas no campo da assistência pré-natal por aplicativos móveis.

Assim, o objetivo do estudo foi mapear os aplicativos móveis de pré-natal descritos e/ou avaliados na literatura científica, em um recorte temporal.

## MÉTODO

### Tipo de Estudo

Estudo de revisão de escopo conduzido a partir das recomendações do The Joanna Briggs Institute (JBI)^(11)^ e conforme as recomendações da diretriz *Preferred Reporting Items for Systematic reviews and Meta-Analyses extension for Scoping Reviews* (PRISMA-ScR). O protocolo do estudo foi publicado na *Open Science Framework*: https://doi.org/10.17605/OSF.IO/8MC96.

### Desenvolvimento da Questão de Pesquisa

Foi utilizada a estrutura mnemônica PCC^(12)^: P (população) – gestantes e profissionais de saúde; C (conceito) - descrição e/ou avaliação de aplicativos móveis; C (contexto) – atividades de saúde relacionadas ao pré-natal. Elaborada a pergunta norteadora: Quais os aplicativos móveis descritos e/ou avaliados na literatura científica sobre atividades de saúde relacionadas ao pré-natal a gestantes e/ou profissionais de saúde?

A busca foi realizada em outubro de 2023.

### Critérios de Elegibilidade

Considerando as atualizações das políticas de saúde na atenção à gestante, foi definido um recorte temporal para o estudo. Foram incluídas pesquisas publicadas de 2017 a 2022, em língua inglesa, portuguesa ou espanhola, disponíveis na íntegra e que apresentassem descrição e/ou avaliação de aplicativos móveis (para *smartphone*) no contexto do pré-natal, a usuários profissionais de saúde ou gestantes.

### Estratégia de Pesquisa

A seleção da amostra deu-se a partir do portal de periódicos da Coordenação de Aperfeiçoamento de Pessoal de Nível Superior (CAPES), por meio do serviço de VPN (*Virtual Private Network*) da Universidade Estadual Paulista (Unesp), rede unespNET, e na busca por artigos nas bases/portais Literatura Latino-Americana e do Caribe em Ciências da Saúde (Lilacs) via Biblioteca Virtual em Saúde (BVS), *Web of Science* (WoS), *National Library of Medicine* (MEDLINE), *Cochrane Database of Systematic Reviews* (COCHRANE), Scopus e *Current Nursing and Allied Health Literature* (CINAHL)(Quadro 1).

**Quadro 1:** Estratégia de busca nas bases de dados – Botucatu, SP, Brasil, 2023.

Base	Estratégia de busca
**Lilacs (via BVS)**	*((“prenatal care”) AND (“Mobile Applications”) AND (la “en” OR “pt” OR “es”))*
**Web of Science**	*(ALL= (“Mobile Applications”)) AND ALL= (“Prenatal care” )*
**MEDLINE**	*“mobile applications” [All Fields] AND “prenatal care” [All Fields]*
**Cochrane**	*(“Mobile Applications”): ti,ab,kw AND (“Prenatal care”): ti,ab,kw*
**SCOPUS**	*“mobile applications”* and *“prenatal care”*
**CINAHL**	*(“mobile applications” or apps or “mobile apps”) AND (“prenatal care” or “pre-natal care” or “perinatal care”)*
**Banco de teses e dissertações da CAPES**	*(“prenatal care”) AND (“Mobile Applications”)*

Para organizar a estratégia de busca, os termos de vocabulário controlado foram selecionados a partir dos Descritores em Ciências da Saúde (DeCS), *Medical Subject Headings Section* (MeSH) e CINAHL *Headings*, combinados com operadores booleanos. Foi realizada busca no Banco de Teses e Dissertações da CAPES (Quadro 1).

### Seleção da Fonte de Evidência

Os títulos e os resumos das pesquisas foram lidos e analisados para identificar os potencialmente elegíveis para o estudo. A fase seguinte envolveu a leitura na íntegra de cada uma das pesquisas selecionadas para: a) confirmar a pertinência à pergunta de revisão e, em caso positivo, b) extrair os dados de interesse, última fase.

### Extração, Análise e Apresentação dos Resultados

A apresentação dos resultados foi realizada por meio de quadros sintéticos e em formato descritivo, conforme as recomendações da diretriz PRISMA-ScR.

O processo de seleção é apresentado, conforme o fluxograma PRISMA-ScR^(13,14)^(Figura 1).

**Figura 1:** Fluxograma PRISMA-ScR de seleção de estudos e processo de inclusão na revisão de escopo – Botucatu, SP, Brasil, 2023.

A extração foi conduzida por dois revisores, os casos de desacordo eram resolvidos por um terceiro revisor. Para a extração dos dados foi utilizado um instrumento adaptado do formulário recomendado pelo JBI^(11)^, com as informações: Autor/ano/país/natureza do estudo, Título/objetivo do trabalho, Nome do aplicativo/idioma/sistema operacional/situação (desenvolvimento ou em uso), Tema central do aplicativo/público alvo.

Os dados extraídos foram mapeados utilizando o software Atlas.ti^(15)^, para organização das informações e processo analítico. Esses foram expressos em quadros sintéticos, discutidos de forma descritiva e comparados aos achados de outros trabalhos correlatos.

Conforme a Figura 1, obteve-se um retorno de 554 pesquisas (549 artigos, 2 dissertações e 3 teses). Títulos e resumos foram lidos, 364 (362 artigos e 2 teses) excluídos, e feito download de 190 trabalhos (187 artigos: Lilacs via BVS – 34, SCOPUS – 50, MEDLINE – 28, WoS – 6, CINAHL – 44 e COCHRANE – 25; 1 tese e 2 dissertações) elegíveis para leitura integral. Excluídas as duplicatas (61), procedeu-se à leitura integral de 129 trabalhos (126 artigos, 2 dissertações e 1 tese).

Nessa etapa, 84 estudos foram excluídos por não tratarem de aplicativos para uso em *smartphones* ou abordarem intervenções utilizando aplicativos já descritos em outros trabalhos analisados/incluídos na revisão. Foi analisada também a lista de referência dos estudos incluídos (adicionados 3 artigos e 1 dissertação).

## RESULTADOS

Aplicados os critérios, após leitura e análise crítica das publicações, foram incluídos 49 estudos, sendo 45 artigos, 3 dissertações e 1 tese, publicados entre 2017 e 2022, predominantemente em inglês (79,6%) e português (20,4%). Quanto ao país de origem, foram desenvolvidos por pesquisadores provenientes das Américas (44,8%) (Brasil 11, Canadá 1, EUA 10, República Dominicana 1), da Ásia (24,4%) (Indonésia 1, China 1, Singapura 71, Japão 1, Índia 1, Irã 2, Israel 1, Malásia 1, Omã 1 República da Coréia 1, Taiwan 1), da Europa (20,4%) (Alemanha 1, Reino Unido 2, Holanda 2, Dinamarca 1, Irlanda 1, Reino Unido 1, Itália 1, Noruega 1), da África (6,1%) (Nigéria 1, Quênia 1, Tanzânia 1) e da Oceania (2%) (Austrália 1).

Foram identificados os desenhos de pesquisa: ensaios clínicos randomizados (22,4%), estudos metodológicos (24,4%), estudos descritivos (20,4%), protocolos para estudo clínico (8,1%), estudos de coorte (8,1%), ensaios clínicos não randomizados (4%) e outros desenhos de pesquisa (12,2%) (estudo experimental, exploratório, métodos mistos, natureza aplicada, observacional, qualiquantitativo).

Foram organizadas categorias conceituais, segundo a finalidade do aplicativo móvel apresentado nos estudos: educação em saúde/autocuidado (46,9%), monitoramento clínico (28,5%), para uso de profissionais (16,3%), pesquisa/coleta de dados (6,1%) e ensino profissional (2%).

A partir dos resultados dos estudos analisados, esses são apresentados em Quadros por caraterísticas dos aplicativos móveis para fins de: letramento, autocuidado e educação em saúde de gestantes e intervenção (Quadro 2); monitoramento clínico (acompanhamento, registro, telemonitoramento, consulta, acompanhamento de patologias e condições clínicas – diabetes mellitus gestacional, sífilis, prematuridade, aspectos psicológicos, etc.) e intervenção (Quadro 3); uso por profissionais da saúde, relacionado ao cuidado de gestantes (Quadro 4); e pesquisa/coleta de dados ou recrutamento de gestantes para pesquisas, intervenção e ensino profissional (Quadro 5).

**Quadro 2:** Caraterísticas dos aplicativos móveis para fins de letramento, autocuidado e educação em saúde de gestantes e intervenção – Botucatu, SP, Brasil, 2023.

Autor/ano/país Natureza do estudo	Título/objetivo	Nome do aplicativo/idioma/sistema operacional/situação (desenvolvimento ou em uso)	Tema central do aplicativo/público-alvo
Kim et al., 2018^(16)^, Estados Unidos, Pesquisa metodológica/produção tecnológica.	*Smartphone-based prenatal education for parents with preterm birth risk factors*/Desenvolver um aplicativo móvel educacional para futuros pais diagnosticados com fatores de risco para parto prematuro, utilizar o aplicativo móvel e testar a viabilidade do conceito em um grupo de pais expectantes diagnosticados com risco de parto prematuro.	*Preemie Prep for Parents* (P3)/inglês/Android e iOS/Desenvolvimento e disponibilidade de uso para avaliação	Educação em saúde/Pais em risco de parto prematuro
Silva et al., 2019^(17)^, Brasil, Estudo descritivo.	Adaptação transcultural do aplicativo *Zero Mothers Die* para dispositivos móveis no Brasil: contribuições para a saúde digital com abordagens do cuidado centrado na e-gestante/Descrever o processo de adaptação do aplicativo para combate à mortalidade materna *Zero Mothers Die*, desenvolvido na Europa, ao contexto brasileiro, com metodologia de escuta individualizada das gestantes e mães usuárias em hospital referência de alta complexidade e de ensino.	*Zero Mothers Die/*Inglês e francês/Android/Uso (Adaptação transcultural para o Brasil)	Educação em saúde/Gestantes, mães de crianças de 0 a 1 ano e profissionais de saúde.
Cawley et al., 2020^(18)^, Estados Unidos, Estudo de coorte retrospectivo.	*Effect of a Health System-Sponsored Mobile App on Perinatal Health Behaviors: Retrospective Cohort Study*/Avaliar se o uso do aplicativo *Circle* durante o período pré-natal estava associado a melhores resultados de saúde e comportamento de saúde entre grávidas e pós-parto.	*Circle*/inglês/iOS/Uso	Gestação e cuidados infantis baseados em evidência/Gestantes e usuários da *Providence St. Joseph*.
Souza et al., 2021^(8)^, Brasil, Ensaio clínico controlado randomizado.	Eficácia de aplicativo móvel na adesão de gestantes às consultas de pré-natal: ensaio clínico randomizado/Avaliar a eficácia de um aplicativo móvel para celular na adesão de mulheres grávidas às consultas de pré-natal.	Gestação Saudável/ português/Android/Uso	Conteúdo educacional em contexto gestacional/Gestantes e profissionais de saúde.
Kennely et al., 2018^(19)^, Irlanda Ensaio controlado randomizado.	*Pregnancy Exercise and Nutrition With Smartphone Application Support*/Avaliar o efeito de um pacote de estilo de vida saudável (uma intervenção de mudança de comportamento pré-natal apoiada por tecnologia de aplicativo de smartphone) na incidência de diabetes mellitus gestacional em mulheres com sobrepeso e obesidade.	*Healthy lifestyle package*/ Inglês/Android e iOS/Uso	Exercício e nutrição na gravidez/Gestantes
Halili et al., 2018^(20)^, Canadá, Pesquisa qualitativa descritiva.	*Development and pilot evaluation of a pregnancy-specific mobile health tool: a qualitative investigation of SmartMoms Canada/R*ealizar uma exploração preliminar das atitudes das mulheres em relação ao aplicativo *SmartMoms Canada* como uma fonte confiável de informações e capacidade de abordar comportamentos de estilo de vida relacionados ao ganho de peso gestacional.	*SmartMoms Canada*/ inglês e francês/Android e iOS/Desenvolvimento	Monitoramento de ganho de peso gestacional/Gestantes
Bush et al., 2017^(21)^, Estados Unidos, Estudo de Caso.	*Impact of a Mobile Health Application on User Engagement and Pregnancy Outcomes Among Wyoming Medicaid Members*/Avaliar se um aplicativo móvel pode alinhar o comportamento do paciente em torno do pré-natal às melhores práticas, melhorando assim os resultados da gravidez.	WYhealth Due Date Plus/Inglês/-------- Uso	Rastreio de fatores de risco gestacional e cuidado/Gestantes
Nørgaard et al., 2017^(22)^, Dinamarca, Estudo de Coorte.	*Use of the smartphone application Pregnant with Diabetes*/Avaliar o uso do aplicativo *Pregnant with Diabetes* local, nacional e internacionalmente.	*Pregnant with Diabetes*/ inglês/ Android e iOS/--------	Educação em saúde para gestantes com diabetes/ Mulheres grávidas com diabetes preexistente
van Beukering et al., 2019^(23)^, Holanda, Estudo de métodos mistos.	*Usability and Usefulness of a Mobile Health App for Pregnancy-Related Work Advice: Mixed-Methods Approach*/Avaliar a usabilidade do aplicativo *Pregnancy and Work* e a utilidade percebida.	*Pregnancy and Work/* holandês e inglês/------- Desenvolvimento (Avaliação de usabilidade)	Aconselhamento de trabalho na gestação/Gestantes
Shorey et al., 2019^(24)^, Cingapura, Estudo de métodos mistos.	*Effectiveness of a Technology-Based Supportive Educational Parenting Program on Parental Outcomes (Part 1): Randomized Controlled Trial*/Avaliar a eficácia de um programa parental educacional de apoio baseado em tecnologia (SEPP) sobre os resultados dos pais durante o período perinatal em casais.	SEEP/ Inglês/ ---------/--------	Intervenção programa educacional parental, Educação em saúde e acompanhamento/Casais
Kawasaki et al., 2021^(25)^, Japão, Protocolo de Estudo controlado randomizado.	*Protocol for an interventional study to reduce postpartum weight retention in obese mothers using the internet of things and a mobile application: a randomized controlled trial (SpringMom)*/Avaliar se a mudança de peso em mulheres obesas aos 12 meses pós-parto em comparação com antes da gravidez se torna menor com uma intervenção utilizando dispositivos com internet das coisas e aplicativos móveis durante a gravidez até 1 ano pós-parto em comparação com e sem intervenção	*Calo Mama Ninsanpu Plus*/----- Android e iOS/ Uso	Monitoramento de ganho de peso gestacional/Gestantes e puérperas
Carrilho et al., 2019^(26)^, Brasil, Estudo observacional, exploratório e descritivo.	Percepção de Gestantes sobre a Interface do Plano de Parto no Aplicativo “Meu Pré-Natal”: Estudo de Validação Observacional/Avaliar a percepção de gestantes sobre a comunicabilidade da elaboração do plano de parto por meio de um aplicativo móvel.	*My prenatal care*/ inglês, português e espanhol/Androide iOS/ Uso	Educação em saúde no pré-natal, parto e puerpério/Gestantes
Moulaei et al., 2021^(27)^, Irã, Estudo descritivo-aplicado/produção tecnológica.	*The Development and Usability Assessment of an mHealth Application to Encourage Self-Care in Pregnant Women against COVID-19*/Desenvolver e avaliar um aplicativo de autocuidado para fornecer autocuidado para gestantes contra a COVID-19.	*Self-Care in Pregnant Women against COVID-19*/ Inglês/----------- Desenvolvimento (prototipagem)	Autocuidado na gravidez contra o COVID-19/Mães e gestantes
Yee et al., 2021^(28)^, Estados Unidos, Estudo prospectivo e qualitativo.	*Patient and Provider Perspectives on a Novel Mobile Health Intervention for Low-Income Pregnant Women With Gestational or Type 2 Diabetes Mellitus*/Coletar *feedback* da SweetMama de pacientes e profissionais de saúde, que informassem as modificações necessárias para melhorar a usabilidade, funcionalidade e acessibilidade do aplicativo.	*SweetMama*/ inglês/----------- desenvolvimento (avaliação de usabilidade de protótipo)	Educação em saúde sobre diabetes mellitus tipo 2 e diabetes gestacional/Mulheres grávidas com Diabetes Mellitus Gestacional e Diabetes Mellitus 2
Lee et al., 2022^(29)^, República da Coréia, Estudo prospectivo e qualitativo.	*Self-Care Mobile Application for South Korean Pregnant Women at Work: Development and Usability Study*/Desenvolver um aplicativo de intervenção baseado em dispositivos móveis para mulheres grávidas coreanas no trabalho e testar sua usabilidade e efeitos preliminares para aprimorar suas práticas de autocuidado.	SPWW/Inglês/ Android/Desenvolvimento e usabilidade	Autocuidado na gravidez/Gestantes
Al Hashmi et al., 2022^(30)^, Omã, Ensaio Clínico.	*Development, feasibility and acceptability of a self-efficacy-enhancing smartphone application among pregnant women with gestational diabetes mellitus: single-arm pilot clinical trial*/Avaliar a viabilidade e a aceitabilidade de um aplicativo para *smartphone*, o SEESPA, entre gestantes com diabetes mellitus gestacional.	SEESPA/ Árabe e inglês/ Android e iOS/ Desenvolvimento	Gestão e controle de diabetes mellitus gestacional/Mulheres com diabetes mellitus gestacional
Sidik et al., 2020^(31)^, Malásia, Protocolo de estudo clínico controlado randomizado por cluster.	*KEPT-app trial: a pragmatic, single-blind, parallel, cluster-randomised effectiveness study of pelvic floor muscle training among incontinent pregnant women: study protocol*/Avaliar a eficácia do aplicativo de orientação do aplicativo KEPT no tratamento da incontinência urinária em gestantes.	KEPT/ ----/----/ Desenvolvimento	Treinamento de músculos do assoalho pélvico/Gestantes e mulheres
Chang et al., 2022^(32)^. Taiwan, Estudo controlado randomizado.	*Efficacy of a Smartphone Application to Promote Maternal Influenza Vaccination: A Randomized Controlled Trial*/Avaliar a eficácia do aplicativo para promover a vacinação materna contra a doença influenza.	*Smartphone Application to Promote Maternal Influenza Vaccination*/ ----/----/ Uso	Vacinação contra gripe/Gestantes
Dudley et al., 2019^(33)^, Estados Unidos, Estudo controlado randomizado.	*Factors associated with referring close contacts to an app with individually-tailored vaccine information*/Determinar quais os fatores associados a uma maior probabilidade de grávidas referirem os seus contatos próximos a esta aplicação educativa.	*MomsTalkShots* ----/----/ Uso para intervenção	Vacinação materna e infantil/Gestantes
Karamolahi et al., 2021^(34)^, Irã, Ensaio clínico randomizado.	*Efficacy of mobile app-based training on health literacy among pregnant women: A randomized controlled trial study*/Determinar o efeito do treinamento baseado em aplicativos móveis na alfabetização em saúde entre mulheres grávidas.	------- ------- Android/ Uso	Educação em saúde /Gestante
Queiroz et al., 2021^(35)^, Brasil, Natureza aplicada, metodológica de abordagem qualitativa.	Avaliação do aplicativo “Gestação” na perspectiva da semiótica: o olhar das gestantes/Avaliar o aplicativo GestAção, a partir do olhar de gestantes, à luz da Semiótica.	GestAção/ Português Android/ Uso para avaliação	Acompanhamento gestacional/Gestantes
Barbosa, 2021^(36)^, Brasil, Desenvolvimento tecnológico.	Desenvolvimento de um Aplicativo Móvel Para Acompanhamento de Gestantes No Pré-Natal, Com Ênfase Na Covid-19/Desenvolver um aplicativo móvel para acompanhamento de gestantes no pré-natal, com ênfase na COVID-19.	INFOGEST no Pré-Natal/ Português/Android/ Desenvolvimento	Educação em saúde da gestante e COVID-19 na gestação/Gestantes
Chiodi, 2020^(37)^, Brasil, Pesquisa aplicada, delineamento metodológico.	Desenvolvimento e avaliação de tecnologia *m-health* direcionada às gestantes com risco para nascimento pré-termo: uma expressão do *deginer thinking.*/Desenvolver e avaliar um protótipo de aplicativo direcionado às gestantes que apresentam risco para o nascimento pré-termo.	Será prematuro?/ Português/ Android e iOS/ Desenvolvimento	Risco para nascimento pré-termo/Gestantes

**Quadro 3:** Características dos aplicativos móveis para fins de monitoramento clínico e intervenção – Botucatu, SP, Brasil, 2023.

Autor/ano/país Natureza do estudo	Título/objetivo	Nome do aplicativo/idioma/sistema operacional/situação (desenvolvimento ou em uso)	Tema central do aplicativo/público-alvo
Miremberg et al., 2018^(38)^, Israel, Estudo controlado randomizado	*The impact of a daily smartphone-based feedback system among women with gestational diabetes on compliance, glycemic control, satisfaction, and pregnancy outcome: a randomized controlled trial*/Analisar o impacto da introdução de um *feedback* diário baseado em *smartphone* e plataforma de comunicação entre pacientes com diabetes mellitus gestacional e seus médicos, na adesão do paciente, controle glicêmico, resultado da gravidez e satisfação do paciente.	Glucosebuddy/ inglês/ Android e IOS/ Uso	Acompanhamento da glicemia/Pessoas com diabetes
Silva et al., 2021^(39)^, Brasil, Produção Tecnológica.	Aplicativo web para acompanhamento de gestantes e puérperas: produção tecnológica/Desenvolver o processo de produção de um protótipo de aplicativo web para o acompanhamento de gestantes e puérperas.	Gestar *care*/ Português/ Android e iOS/ Desenvolvimento e uso	Saúde materno-infantil e consulta online/Profissionais de saúde, gestantes e puérperas
Doherty et al., 2018^(40)^, Irlanda e Reino Unido, Pesquisa Clínica.	*A Mobile App for the Self-Report of Psychological Well-Being During Pregnancy (BrightSelf): Qualitative Design Study*/Explorar as questões e desafios que envolvem o uso de telefones celulares para o autorrelato de bem-estar psicológico durante a gravidez.	*BrightSelf*/ inglês/ Android e iOS/ Uso	Avaliação e monitoramento de depressão e humor durante o pré-natal/Gestantes
Hantsoo et al., 2018^(41)^, Estados Unidos, Estudo controlado randomizado.	*A Mobile Application for Monitoring and Management of Depressed Mood in a Vulnerable Pregnant Population*/Avaliar o impacto de um aplicativo móvel de rastreamento e alerta de humor no envolvimento do paciente e na prestação de cuidados de saúde em um ambiente obstétrico entre mulheres de grupos étnico raciais minoritários com baixa renda que apresentavam sintomas depressivos.	*Mood Tracking and Alert* (MTA)/inglês/ Android e iOS/ Uso	Saúde mental/População em geral
Marko et al., 2019^(42)^, Estados Unidos, Estudo Controlado.	*A Mobile Prenatal Care App to Reduce In-Person Visits: Prospective Controlled Trial*/Testar a eficácia de um aplicativo móvel de atendimento pré-natal para facilitar um agendamento de visitas presenciais reduzido para gestações de baixo risco, mantendo a satisfação do paciente e do profissional.	*Babyscripts*/ inglês/ iOS/Uso	Conteúdo educacional/ Gestantes
Sales et al., 2019^(43)^, Brasil, Pesquisa metodológica.	Desenvolvimento e avaliação de um aplicativo para o controle da sífilis em gestantes/Desenvolver e avaliar um aplicativo para o controle da sífilis em gestantes.	SELP/ português/ Android e iOS/Desenvolvimento	Prevenção de sífilis/População em geral e profissionais de saúde
Borgen et al., 2019^(44)^, Noruega, Ensaio clínico controlado randomizado.	*Effect of the Pregnant+ smartphone application in women with gestational diabetes mellitus: a randomised controlled trial in Norway*/Avaliar o efeito do aplicativo *Pregnant+* no nível de glicose de 2 horas do teste de tolerância à glicose oral pós parto de rotina em mulheres com diabetes mellitus gestacional.	*Pregnant+*/ ------- inglês/ uso	Manejo de diabetes gestacional/Gestantes
Xydopoulos et al., 2019^(45)^, Reino Unido, Viabilidade econômica - Estudo coorte.	*Home blood-pressure monitoring in a hypertensive pregnant population: cost-minimization study*/Realizar uma análise econômica da saúde do aplicativo *HBPM* em comparação com o acompanhamento tradicional em gestantes hipertensas.	*HBPM*/ inglês/-----/ Desenvolvimento	Educação em saúde e monitoramento de saúde/Gestantes
Müller et al., 2020^(46)^, Alemanha, Protocolo de Ensaio clínico randomizado controlado.	*Effectiveness and cost-effectiveness of an electronic mindfulness-based intervention (eMBI) on maternal mental health during pregnancy: the mindmom study protocol for a randomized controlled clinical trial*/Avaliar a eficácia clínica e custo-benefício de aplicativo desenvolvido por uma equipe interdisciplinar de ginecologistas, psicólogos e parteiras, ensinando as gestantes a lidar com o estresse, a ansiedade relacionada à gravidez e os sintomas depressivos.	*Mindmom* / ----------- ----------- Uso	Saúde mental materna/Gestantes
Cramer et al., 2018^(47)^, Estados Unidos, Estudo experimental - Pesquisa participativa baseada na comunidade.	*The feasibility and promise of mobile technology with community health worker reinforcement to reduce rural preterm birth*/Avaliar a viabilidade de uma intervenção de plataforma de *smartphone* combinada com o reforço de agentes comunitários de saúde em mulheres grávidas rurais; Obter dados sobre a intervenção nos resultados do parto, ativação do paciente e adesão aos cuidados médicos; e Avaliar as implicações financeiras da intervenção usando o retorno do investimento	PTP/ inglês e espanhol/ -------/ Desenvolvimento (estudo de viabilidade – piloto)	Educação em saúde gestacional/Gestantes
van den Heuvel et al., 2019^(48)^, Holanda, Observacional Prospectivo.	*SAFE@HOME - Feasibility study of a telemonitoring platform combining blood pressure and preeclampsia symptoms in pregnancy care*/Avaliar a aceitabilidade do paciente do telemonitoramento usando o aplicativo e o monitor de pressão arterial e revisar a infraestrutura interna para analisar todas as medidas observadas.	Luscii/ Holandês/ iOS/uso	Telemonitoramento da pressão arterial no pré-natal/Gestantes e profissionais da saúde
Zuccolo et al., 2021^(49)^, Brasil, Protocolo de ensaio clínico randomizado.	*A smartphone-assisted brief online cognitive-behavioral intervention for pregnant women with depression: a study protocol of a randomized controlled trial*/Avaliar a eficácia do *Motherly*, para terapias cognitivo-comportamentais no tratamento da depressão materna.	*Motherly*/ português Android/Uso	Educação em saúde da gestante e bebê (saúde mental – ativação comportamental)/Gestantes e mães
Musyoka et al., 2019^(50)^, Quênia, Estudo metodológico, exploratório experimental.	*A 24-hour ambulatory blood pressure monitoring system for preeclampsia management in antenatal care*/Implementar um sistema de monitoramento ambulatorial de pressão arterial 24 horas para gestantes usando um *smartwatch* em conjunto com um aplicativo móvel, para auxiliar no gerenciamento da pré-eclâmpsia, baseado em uma arquitetura de Internet das Coisas.	*Blood pressure monitor*/ Inglês/Android/ Desenvolvimento	Monitoramento de pressão arterial/Gestantes e profissionais

**Quadro 4:** Características dos aplicativos móveis para uso de profissionais da saúde vinculados ao cuidado de gestantes e intervenção –Botucatu, SP, Brasil, 2023.

Autor/ano/país Natureza do estudo	Título/objetivo	Nome do aplicativo/idioma/sistema operacional/situação (desenvolvimento ou em uso)	Tema central do aplicativo/público-alvo
Gbadamosi et al., 2018^(51)^, Nigéria, Estudo de demonstração.	*A Patient-Held Smartcard With a Unique Identifier and an mHealth Platform to Improve the Availability of Prenatal Test Results in Rural Nigeria: Demonstration Study*/Descrever o desenvolvimento e implantação de uma plataforma *mHealth* integrada e capaz de capturar informações de saúde, incluindo resultados de exames laboratoriais, e criptografá-las em um cartão inteligente do paciente, que pode ser lido no ponto de entrega sem a necessidade de conexão com a Internet.	*Vitira Health*/inglês/ Android/Desenvolvimento (restrito a usuários autorizados)	Leitura de código QR de Smartcard e exibição de resultados de exames pré-natais/Pacientes autorizados
Borsari et al., 2018^(52)^, Itália, Estudo de viabilidade.	*An Innovative Mobile Health System to Improve and Standardize Antenatal Care Among Underserved Communities: A Feasibility Study in an Italian Hosting Center for Asylum Seekers*/Apresentar os resultados de um estudo piloto de avaliação de funcionalidade, viabilidade e taxa de aceitação do sistema de saúde móvel no fornecimento de cuidados pré-natais a uma população migrante hospedada dentro do maior centro de recepção europeu.	PANDA/ Inglês e francês/ Android/ Desenvolvimento	Cuidado pré-natal centrado na Avaliação Diagnóstica da Gravidez e do Recém-nascido /Profissionais de saúde (médicos/as, enfermeiros/as, parteiras, agentes comunitários de saúde)
Shah et al., 2019^(53)^. Índia. Estudo de Viabilidade.	*High uptake of an innovative mobile phone application among community health workers in rural India: An implementation study*/Avaliar a aceitação, viabilidade e eficácia do ImTeCHO, no ambiente de trabalho pessoal.	ImTeCHO/ Indiano/ Android/ Uso	Gerenciamento de trabalho no acompanhamento de gestantes e crianças/Profissionais, *Accredited social health activist* - ASHA (semelhante aos agentes comunitários de saúde)
Bonnell et al., 2018^(54)^, República Dominicana, Estudo de viabilidade.	*Community Health Workers and Use of mHealth: Improving Identification of Pregnancy Complications and Access to Care in the Dominican Republic*/Avaliar a viabilidade e aceitabilidade de uso de tecnologia móvel de saúde por agentes comunitários de saúde para melhorar a identificação de complicações na gravidez e o acesso aos cuidados para mulheres grávidas.	*Poimapper* (aplicativo móvel e portal de gerenciamento de dados)/------/Android e iOS/Uso	Identificação de complicações na gravidez e acesso a cuidados para mulheres grávidas /Profissionais, Agentes comunitários de saúde
Hackett et al., 2018^(55)^, Tanzânia, Estudo randomizado por cluster.	*Impact of smartphone-assisted prenatal home visits on women’s use of facility delivery: Results from a cluster-randomized trial in rural Tanzania*/Avaliar o impacto de um aplicativo para Agentes comunitários de saúde na utilização de serviços de parto por mulheres na Tanzânia rural.	SUSTAIN/ ----/----/ Uso	Gestão e acompanhamento de visitas domiciliares de gestantes/Agentes comunitários de saúde (ACS)
Liu e Wang, 2021^(56)^, China, Estudo clínico de intervenção.	*Application of Smart Mobile Medical Services in Maternal Health Care Management*/Padronizar a gestão da saúde das gestantes, melhorar o nível de saúde das gestantes e melhorar o desfecho da gravidez com o auxílio do aplicativo móvel.	*Health Assistant*/ -------/------- Uso	Gestão de educação em saúde/Gestante
Delmaifanis et al., 2021^(57)^, Indonésia, Estudo conceitual e desenvolvimento de aplicativo.	*mHealth Conceptual Model for Providing Quality Antenatal Care in Health Centers during the Coronavirus Disease 2019 Pandemic*/Construir o conceito de *mHealth* para melhorar a qualidade do cuidado pré-natal, para adicionar recursos que apoiem os trabalhadores de serviços durante a pandemia de COVID-19.	-------/ inglês/ ------- desenvolvimento	Acompanhamento e cuidado pré-natal durante a pandemia/Parteiras, médicos e gestantes
Fonseca, 2020^(58)^, Brasil, Estudo documental e metodológico.	Revisão e Atualização de Subconjuntos Terminológicos CIPE® para Saúde da Mulher, Pré-Natal e Pós-Parto e Proposta de Construção de Aplicativo para dispositivos Móveis/Descrever o processo de revisão e atualização e apresentar os subconjuntos terminológicos de Saúde da Mulher e de Pré-natal e Pós-parto, segundo a CIPE® 2017 e propor a modelagem para a construção de um aplicativo para dispositivos móveis, para disponibilizar os Subconjuntos Terminológicos.	CIPE-APS/ Português/ Android e iOS/ Desenvolvimento	Subconjuntos terminológicos de Saúde da Mulher e de Pré-natal e Pós-parto, segundo a CIPE® /Enfermeiros, professores de enfermagem e estudantes de enfermagem

**Quadro 5:** Características dos aplicativos móveis para pesquisa/coleta de dados ou recrutamento de gestantes para pesquisas, intervenção e ensino em enfermagem obstétrica – Botucatu, SP, Brasil, 2023.

Autor/ano/país Natureza do estudo	Título/objetivo	Nome do aplicativo/idioma/sistema operacional/situação (desenvolvimento ou em uso)	Tema central do aplicativo/público-alvo
Krishnamurti et al., 2021^(59)^, Estados Unidos, Estudo de coorte prospectivo.	*Use of a Smartphone App to Explore Potential Underuse of Prophylactic Aspirin for Preeclampsia*/Avaliar o potencial de subutilização do tratamento com aspirina em baixas doses e os motivos da subutilização usando dados de um aplicativo de pré-natal.	*My Healthy Pregnancy* (MHP)/inglês/ Android e iOS/ Uso	Conteúdo educacional em contexto gestacional/Gestantes
Vignato et al., 2019^(60)^, Estados Unidos, Estudo transversal.	*Using Mobile Health Applications for the Rapid Recruitment of Perinatal Women/*Descrever como duas equipes de pesquisa dos Estados Unidos utilizaram um aplicativo móvel destinado a mulheres grávidas para recrutar participantes para um estudo transversal e um ensaio clínico	*Ovia Pregnancy*/ inglês/Android e iOS/ Uso	Educação em saúde gestacional/Gestantes
Keedle et al., 2018^(61)^, Austrália, Pesquisa qualitativa	*The Design, Development, and Evaluation of a Qualitative Data Collection Application for Pregnant Women*/Descrever o desenvolvimento e avaliação de um aplicativo móvel para coletar dados qualitativos.	*myVBACapp* /Inglês/ Android e iOS/ Desenvolvimento	Coleta de dados/Mulheres
Amorim, 2022^(62)^, Brasil, Estudo Metodológico.	GRAVIDAPP 2.0: aplicativo educacional para a prática da enfermagem obstétrica no ensino superior/Desenvolver um aplicativo educacional intitulado *gravidapp 2.0* para suporte teórico-prático de discentes da disciplina de Enfermagem Obstétrica (EO) da Escola de Enfermagem Magalhães Barata da Universidade do Estado do Pará (EEMB-UEPA).	Gravidapp 2.0/ Português/Android/ Uso	Ensino de Enfermagem Obstétrica/Alunos, residentes e/ou profissional da Enfermagem Obstétrica

Quanto aos aplicativos para fins de letramento, autocuidado e educação em saúde de gestantes (Quadro 2), identificou-se que quatro desses foram desenvolvidos para a promoção de cuidados no pré-natal ou mesmo para condições específicas como COVID-19 e sífilis. Oito (16,3%) aplicativos foram usados para intervenção, seja na avaliação do uso frente ao monitoramento de condições clínicas (ganho de peso, COVID-19), adesão às consultas e estímulo ao autocuidado com informações sobre estilo de vida saudável.

Seis (12,2%) aplicativos foram avaliados, de forma global ou em uma funcionalidade específica, quanto à aceitabilidade do seu uso para telemonitoramento e quanto à coleta de feedback para melhoria de funcionalidade e acessibilidade do aplicativo. Três (6,1%) foram avaliados quanto à eficácia na promoção de tratamentos, adesão a vacinas, melhores práticas no pré-natal e agendamento de atendimento. Um (2%) aplicativo de combate à mortalidade estava em processo de adaptação transcultural para o Brasil e um (2%) foi avaliado quanto à qualidade do conteúdo.

No Quadro 3, são apresentados os estudos referentes aos aplicativos para monitoramento clínico. Foram identificados três (6,1%) estudos referentes ao desenvolvimento de aplicativos voltados para o acompanhamento e controle de sífilis, e um a identificar requisitos para o aplicativo ideal. Seis (12,2%) estudos realizaram intervenção usando aplicativos e avaliaram diversos aspectos: comunicação paciente-profissional, potencial para monitoramento (diabetes mellitus gestacional, tolerância à glicose oral pós-parto, aspectos psicológicos, pressão arterial combinada com *smartwatch*) e o efeito do uso da tecnologia móvel no monitoramento combinado com reforço de Agentes Comunitários de Saúde (ACS).

Três (6,1%) estudos avaliaram a eficácia no agendamento e quanto ao fornecimento de breves terapias cognitivo-comportamentais para tratar a depressão materna. Outros dois (4%) estudos avaliaram o custo-benefício dos aplicativos quando comparados ao acompanhamento tradicional e a eficácia clínica, e por fim um voltado para avaliar a aceitabilidade de aplicativo para telemonitoramento de pressão arterial.

No Quadro 4, sobre os aplicativos para uso por profissionais, dois (4%) estudos apontaram o desenvolvimento de um aplicativo para captar informações de saúde, acesso aos exames e para auxiliar a assistência durante a pandemia de COVID-19. Três (6,1%) avaliavam a funcionalidade e viabilidade dos aplicativos quando estes eram usados para fornecer cuidados pré-natais ou no auxílio ao trabalho de agentes comunitários de saúde. Um (2%) estudo avaliou o impacto da implantação do aplicativo em desfechos de parto, por meio do uso para comunicação entre ACS e a equipe de saúde, que direcionava o cuidado. Outros dois (4%) estudos padronizaram a assistência, tanto por orientações fornecidas pelo aplicativo, quanto na uniformização da linguagem utilizada entre os profissionais.

No Quadro 5 são apresentados três (6,1%) estudos nos quais foram abordados os aplicativos desenvolvidos ou em uso como meio acessível e a apoiar a coleta de dados para pesquisa científica envolvendo gestantes e um (2%) estudo demonstrou o desenvolvimento e uso de aplicativo para o ensino profissional.

## DISCUSSÃO

Os aplicativos móveis têm apoiado diversas atividades em saúde, no contexto do pré-natal, auxiliando no letramento em saúde, autocuidado, monitoramento clínico pela usuária e pelos profissionais de saúde, bem como para fins de educação em enfermagem.

O letramento em saúde é potencialmente facilitado pelo apoio de aplicativos, no qual seu uso se mostrou positivo para educação parental no pré-natal de gestantes com risco de parto prematuro^(16)^, identificação da necessidade de abordar aspectos psicossociais da gestação de alto risco e preparo para possível situação de internação, parto pré-termo e internação neonatal^(37)^, promoção de autocuidado na pandemia de COVID-19, além de fornecerem informações, orientações acerca de medicações e complicações da COVID-19 na gestação^(27,36)^.

Estudos de intervenção demonstraram que o uso de aplicativos foi eficaz para monitoramento de condições clínicas, favorecendo a comunicação entre equipe de saúde e gestante^(8)^, na adesão das gestantes às consultas pré-natal, aprimoramento de práticas saudáveis, autocuidado durante o pré-natal^(8,18,25)^), letramento em saúde de gestantes no contexto da pandemia^(34)^ e informações personalizadas sobre vacinas^(33)^.

Acerca dos aplicativos já desenvolvidos que estavam sendo avaliados de forma global ou em alguma funcionalidade, os estudos mostraram usabilidade e aceitabilidade positivas. Apresentaram uso positivo para telemonitoramento e oferta de informação sobre índice glicêmico, via tecnologia, em áreas rurais e em países em desenvolvimento^(22)^, facilitando o cuidado materno-infantil de forma interativa e educativa^(35)^, com alta eficácia clínica na saúde mental materna, promovendo apoio psicológico a pais e mães, por meio de fontes confiáveis de informação^(24,30)^ e boa avaliação percebida mesmo quando avaliada apenas a interface para plano de parto^(26)^.

Estudos^(23,26)^ analisaram o feedback a partir de usuárias e foi identificado que o design utilizado influencia a interação destas com o aplicativo. Foram encontrados resultados positivos no uso de interfaces interativas e que oferecem conteúdos com informação à usuária.

Estudos avaliaram a eficácia dos aplicativos quanto ao lembrete de vacinação contra influenza e ao conhecimento das gestantes sobre a vacina, promovendo atitudes positivas em relação à vacinação^(32)^. Além disso, Sidik^(31)^ propôs o desenvolvimento de um aplicativo de acordo com um mapeamento de intervenção, avaliado quanto à eficácia na orientação para tratamento da incontinência urinária em gestantes.

O aplicativo *SweetMama* foi implementado para intervenção móvel de saúde para gestantes de baixa renda com diabetes gestacional, e demonstrou ser de fácil uso, eficiente e potencialmente impactante, projetado para atender às necessidades exclusivas dessa população^(28)^.

Além do desenvolvimento e avaliação dos aplicativos, estudo^(34)^ propôs a adaptação cultural de um aplicativo para combate à mortalidade materna, da língua inglesa para o português do Brasil. O *Zero Mothers Die* é um aplicativo com informação sobre a rotina pré-natal de estabelecimentos de saúde com atividades de ensino, o qual pode oportunizar canais de diálogo com a gestante e atualização do profissional em treinamento^(17)^.

A preocupação com o monitoramento clínico de determinadas condições que podem emergir na gestação ou afetar a gestante de forma direta ou indireta foi destacada em estudos, tanto nas propostas de desenvolvimento e/ou protocolos de ensaio clínico, quanto pelas intervenções utilizando tais ferramentas^(46,49)^.

Destaca-se estudo^(43)^ no qual os autores desenvolveram o aplicativo SELP com informações em texto e vídeo acerca dos sinais, sintomas, causas, riscos e tratamento para sífilis. A tecnologia aplica recursos de mapeamento para localizar postos de saúde, cria alertas à gestante e ao parceiro sobre datas de tratamento e realiza mapeamento da rede de contatos de portadores de sífilis de maneira anônima^(43)^.

Aplicativos móveis são uma importante ferramenta para otimizar tempo e encurtar distâncias. Por serem ferramentas de fácil acesso e de consulta rápida, demonstraram serem eficazes para reduzir visitas presenciais, favorecem atendimento virtual de gestantes e puérperas, e reduzem o tempo de espera por serviços de emergência e profissionais, dados seus recursos de comunicação^(39,42)^. Além da possibilidade de comunicação com um profissional para atendimento, diversos estudos demonstram a eficácia dos aplicativos no telemonitoramento da pressão arterial (PA) de gestantes^(45,48,51)^.

Foi possível promover o monitoramento de PA de gestantes usando um *smartwatch* em conjunto com aplicativo móvel; houve redução do número de consultas ambulatoriais pré-natais por motivos relacionados à PA em comparação com o manejo de acordo com diretrizes locais e economia de custos com o uso do aplicativo^(45,51)^. A solução demonstrou potencial para sua adoção em sistemas de saúde em países em desenvolvimento, dada a simplicidade e acessibilidade do aplicativo^(51)^.

Outro recurso, de envio de alertas para início de medição repetida da PA, combinado a alertas de sintomas autorrelatados de pré-eclâmpsia, também demonstrou relevância clínica^(48)^.

Intervenção de feedback diário e comunicação entre pacientes e equipe de saúde melhorou a adesão do paciente e o controle glicêmico, e reduziu a taxa de tratamento com insulina^(38)^.

Em contrapartida, ao analisar o efeito de uso de um aplicativo para gestante (Pregnant+) em mulheres com diabetes mellitus na Noruega, os resultados apontaram que o uso do aplicativo não apresentou efeito no desfecho principal de monitorização de glicose e redução de taxas^(44)^. Tal achado pode estar relacionado ao fato de a tecnologia não ter sido desenvolvida especificamente para essa finalidade, o que pode comprometer a eficácia da estratégia para o telemonitoramento.

No impacto para saúde mental perinatal, uma ferramenta para o autorrelato do bem-estar psicológico mostrou potencial em permitir uma maior compreensão do bem-estar na gravidez^(40)^. Outro estudo^(41)^ apresentou a necessidade de promoção de terapia cognitivo-comportamental utilizando tecnologia móvel, e mostrou eficácia do aplicativo na melhoria dos serviços de saúde mental na prática obstétrica. Intervenções como esta podem ajudar a identificar precocemente, monitorar e tratar mulheres com alto risco de desenvolver sintomas psiquiátricos durante a gravidez, além de ter potencial para oferecer informações baseadas em evidência científica, auxiliando-as a identificar ansiedades e inseguranças desse período e buscar apoio.

Aplicativos móveis têm um custo de desenvolvimento e manutenção nas plataformas digitais. Por isso, estudos de avaliação de custo-efetividade também estão descritos.

Estudo^(47)^ realizou análise financeira (retorno sobre investimento) na qual um grupo de gestantes (grupo intervenção) recebeu um *smartphone* com internet e acesso a plataforma de pré-natal personalizada (avaliada no estudo) com mensagens de texto automatizadas, função de bate-papo e *hiperlinks* e contato semanal do ACS. O grupo controle recebeu o cuidado pré-natal habitual e materiais educativos impressos. A intervenção obteve avaliação positiva das usuárias e mostrou-se promissora para a adesão aos cuidados pré-natais e a melhorar os resultados do parto em comunidades rurais. A análise de impacto financeiro mostrou resultados positivos de custo-efetividade para a intervenção, produzindo redução de custos de saúde para o grupo de intervenção, em relação ao grupo de controle, com diferença média geral de economia de US$ 1.079 para os participantes da intervenção.

Dada a versatilidade e acessibilidade dos aplicativos, estes também foram utilizados como recursos, para acesso fácil e rápido para recrutar participantes para pesquisa científica envolvendo gestantes^(59)^. O uso de aplicativos para recrutamento de participantes permite dados em tempo real e acesso instantâneo aos dados, além de possibilitar contatar pessoas de vários locais^(61)^, estratégia promissora para o recrutamento de amostras grandes e diversificadas^(60)^.

A versatilidade dos aplicativos tem permitido integrar seu uso com outras ferramentas ou plataformas de saúde, para implementar cuidados no pré-natal e acessar informações da gestante, mesmo em locais com baixa disponibilidade de internet^(61)^ ou durante a pandemia de COVID-19^(57)^, otimizando a qualidade e efetividade dos serviços e a promoção da saúde.

Além de auxiliar na assistência à gestante, aplicativos têm sido utilizados por profissionais de saúde para fornecerem recomendações de cuidado pré-natal, a partir de diretrizes e recomendações de forma estruturada. Alguns resultados são observados, como a melhora da comunicação entre pacientes e cuidadores^(52)^, sistematização das visitas domiciliares e qualificação do atendimento pré-natal e pós-natal, juntamente com o atendimento de casos de maior complexidade, viabilizado pela possibilidade de contato do ACS com a equipe de saúde^(53,54)^.

O uso de *smartphones* como um componente dos pacotes de intervenção voltados para a maternidade segura pode melhorar o apoio e a efetividade da atuação dos ACS^(58)^ e demais profissionais de saúde, melhorando as taxas de adesão do paciente a exames e seguimento no pré-natal, por meio da oferta de informações durante a gestação e o período perinatal^(56)^.

Além dos aplicativos desenvolvidos para oferta de cuidados via telefonia móvel, estudo^(56)^ propôs a construção de um aplicativo para dispositivos móveis para disponibilizar o Subconjunto Terminológico de Saúde da Mulher, Pré-natal e Pós-parto a partir da versão traduzida para o português da Classificação Internacional para a Prática de Enfermagem (CIPE®). Outra pesquisa^(62)^ propôs o desenvolvimento de um aplicativo para o ensino em enfermagem obstétrica.

Essas propostas demonstram um novo perfil de aplicativos móveis voltados a apoiar a assistência prestada por profissionais e a auxiliar estudantes no processo de ensino aprendizagem de habilidades específicas para a enfermagem obstétrica.

### Limitações Do Estudo

Limitações do estudo devem ser consideradas. Foi considerado um recorte temporal de 2017 a 2022, limitando a análise da produção científica a um período determinado. Não foi avaliada a qualidade metodológica dos estudos. Alguns estudos incluídos não apresentavam informações completas dos aplicativos e por vezes as informações de caracterização estavam ausentes, tais como sistema operacional, idioma e situação (desenvolvimento ou em uso). Não foi possível avaliar com critérios a fundamentação científica do conteúdo dos aplicativos; entretanto, destaca-se que a divulgação em publicações científicas torna estes mais confiáveis para serem recomendados/utilizados na atenção ao pré-natal.

### Contribuições para Pesquisas Relacionadas à Saúde

Esta revisão fornece um panorama abrangente dos aplicativos móveis descritos/avaliados na literatura científica, no contexto de acompanhamento da gestante nas diversas situações clínicas no período pré-natal. Favorecem o acesso à informação no contexto do pré-natal por tecnologia móvel, tanto a mulheres grávidas e seus parceiros, a profissionais de saúde e a estudantes. Considerando a divulgação de estudos de revisão de aplicativos que partem da análise das lojas de aplicativos, esta revisão parte dos aplicativos divulgados em produções científicas, o que possibilita maior confiança nas recomendações apontadas. Ademais, este estudo fornece recomendações para o desenvolvimento e a avaliação de tecnologias móveis em saúde.

## CONCLUSÃO

As tecnologias em saúde ganham cada vez mais destaque no cenário global. Por isso, caracterizar os aplicativos móveis utilizados ou idealizados para o acompanhamento da gestante a partir da literatura científica ofereceu uma visão ampliada dessas tecnologias e de suas formas de uso.

Os estudos analisados trouxeram aplicações móveis versáteis e variadas, cujo uso confirma o potencial da inserção da tecnologia móvel nos diversos cenários do cuidado e no monitoramento da saúde das gestantes. Ademais, quer sejam utilizados por profissionais, estudantes da saúde ou pelas próprias gestantes, os aplicativos em tela foram amplamente utilizados nas áreas de letramento, autocuidado, educação em saúde de gestantes, monitoramento clínico e intervenção, ensino em enfermagem obstétrica e para coleta de dados ou recrutamento de gestantes para pesquisas.

Desdobramentos futuros podem ser necessários para que aplicativos possam ser avaliados utilizando instrumentos que considerem aspectos técnicos, segurança, qualidade dessas soluções ou mesmo explorem o potencial dos aplicativos em outras interfaces, do acompanhamento da gestante ao longo do pré-natal, ampliando os resultados apresentados nesse estudo.
